# Acknowledgment to the Reviewers of *JPM* in 2022

**DOI:** 10.3390/jpm13020164

**Published:** 2023-01-17

**Authors:** JPM Editorial Office

**Affiliations:** MDPI AG, St. Alban-Anlage 66, 4052 Basel, Switzerland

High-quality academic publishing is built on rigorous peer review. *JPM* was able to uphold its high standards for published papers due to the outstanding efforts of our reviewers. Thanks to the efforts of our reviewers in 2022, the median time to first decision was 21 days and the median time to publication was 41 days. Regardless of whether the articles they examined were ultimately published, the editors would like to express their appreciation and thank the following reviewers for the time and dedication that they have shown *JPM*:
A. Alexandre TrindadeLaura-María Compañ GabucioAaran LewisLaurent GuyotAaron Blandino OrtizLaurent MachetAatish MahajanLaurie Fields DeRoseAbbas ShafieeLaurie M. ElitAbbas SmileyLaurie MarrauldAbbirami ElangovanLeana Ioana CofaruAbdalkarem AlsharariLee NguyenAbdolhassan KazemiLee Samüel WannAbdollah AllahverdiLehel CsatóAbdolreza JamilianLei GuoAbdorreza Naser-MoghadasiLeila JahangiriAbdulkarim HasanLeili ShahriyariAbdullah AssiriLeire PedrosaAbdullah LaherLeitao SunAbel Emanuel MocaLele ZhuAbhay ShahLena WimmerAbhilasha Kandahalli Venkataranga NayakaLennart HammarströmAbhinav DubeyLenora M. OlsonAbhinava MishraLeo L. TsaiAbhishek KulkarniLeonard BieloryAbhishek KumarLeonardo BrunettiAbraham DibLeonardo ChiattiAbubakr H. MossaLeonardo ErminiAchille AvetaLeonardo Peyré-TartarugaAcke FredericLeonardo Saúl Lino-SilvaAdam D. KennedyLeonardo SpatolaAdam DyerLeonhard MüllauerAdam KernLeopold CurfsAdam WalkowiakLeslie Chavez-GalanAdam WeitemierLeyla KuruAdelina VladLi GunAdina RoceanuLi HeAdolfo Baloira VillarLi OuAdovich S. RiveraLi-An ChuAdriana AlbuLian-Hua HuangAdriana M. CalvoLianwu FuAdriana Maria NeagLídia FeliubadalóAdriana PolanskaLiisa VeerusAdriano FriganovicLijuan FengAdriano MurroneLilach SoreqAdriano Polican CienaLiliana G. CiobanuAftab AlamLiliana MajkowskaAgam BansalLiliana RockenbachAgilandeeswari LoganathanLiliya S. LogoydaAgnieszka FogelLilya DzhemilevaAgnieszka LasotaLin ChengAgnieszka Lecka-AmbroziakLina ZuccatostaAgnieszka Owczarczyk-SaczonekLinchen HeAgnieszka PotęgaLincoln Faria Da SilvaAgnieszka Strzelecka-KiliszekLinda HersheyAgnius LiutkeviciusLinda ReisAgustín Díaz-ÁlvarezLinda SangalliAhmad AfifyLindawati S. KusdhanyAhmad M. MansourLing HeAhmad MirzaLing SunAhmad Rusdan Handoyo UtomoLing TangAhmed AbdelhakLing XuAhmed Abu-AwwadLing YinAhmed BahgatLinpeng Andrew ShanAhmed ElamragyLinshuai ZhangAhmed ElsawahLinu Sara GeorgeAhmed Ezat El ZowalatyLinyan MengAhmed Hussieny ElbarbaryLinyi WangAhmet AltanLirong WangAhmet AslanLisa ArgnaniAhmet AyazLisa ChristAhmet Emin TopalLisa GouldAhmet TahraLisa J. UnderlandAhmet TanhanLisa WarnerAida MetoLisse Angarita DavilaAidel SalihLivio VitielloAiden EblimitLiwei ChenAikaterini MastorakiLi-Wen LeeAiko NagaiLixin ChengAinhoa Plaza-ZabalaLixin RuiAinhoa Robles-MezcuaLiza LeeAjay PalLoknath Sai AmbatiAjitanuj RattanLong Chiau MingAkarsh VermaLoredana BergandiAkash Kumar BhoiLoredana MarcuAkhtar AkhtarLorena CiumărneanAkihiro TanemuraLorenzo AngeliniAkihito TsubotaLorenzo Ferro DesideriAkio OishiLorenzo FranceschettiAkira UmemuraLorenzo MagrassiÁkos LukátsLoretta L. MüllerAkram Hernández-VásquezLourdes GimenoAkriti SrivastavaLubos CipakAkshara RaghavendraLuc FavardAkshay AnandLuc MaroteauxAkshay KumarLuca Di GianfrancescoAkshay PandeyLuca FlesiaAla’a B. Al-TammemiLuca FranchinAlaattin OzenLuca GazziniAlbert Thomas AnastasioLuca GiannellaAlberto Adanero VelascoLuca Giovanni LocatelloAlberto AntonicelliLuca OngaroAlberto CaggiatiLuca PuzziAlberto Francesco CeredaLuca Salvatore De SantoAlberto MaccariLuca SoraciAlberto MellaLuca TestarelliAlberto Molares-VilaLucas WesselAlberto PardoLucatelli PierleoneAlberto SaitaLucia AnsaniAldo PezzutoLucia PirvuAldo RoccaroLucia Princiotta CariddiAlejandro Gómez-MejiaLucía Silva-FernándezAlejandro J. Marquez-LaraLucia SilvestriniAleksandar MiladinovicLucia TricaricoAleksandar RakićLuciana CaenazzoAleksandar StojsavljevićLuciana Maria Malosá SampaioAleksandar SzéchenyiLuciana Teodora RotaruAleksander Jerzy OwczarekLucrezia TognoloAleksander MendykLudek SlavikAleksandr MironovLudmila Danilowicz-SzymanowiczAleksandra AsaturovaLudovic AillotAleksandra DjokovicLudovica Anna CiminiAleksandra Gaworska-KrzemińskaLudovico AbenavoliAleksandra JarońLudovico PedullàAleksandra JoticLuigi Alberto PiniAleksandra KuzanLuigi CerboneAleksandra M. PavlovićLuigi Di TommasoAleksandra SuchaneckaLuigi GianturcoAleksei TikhonovLuigi LoscoAleš TomažičLuigi NapolitanoAlessandra AldieriLuigi SantacroceAlessandra MurriLuigi Segagni LusignaniAlessandra Verzelloni SefLuigi TarantiniAlessandro AlaimoLuigi TroisiAlessandro AntonelliLuigi-alberto PiniAlessandro Baldi AntogniniLuis Abel QuiñonesAlessandro CaputoLuis Andrés López FernándezAlessandro CeliLuís Carlos Lopes-JúniorAlessandro CrosioLuis Cobos-PucAlessandro De SireLuis D’MarcoAlessandro GhezzoLuis FernandezAlessandro MalobertiLuis RodrigoAlessandro MazzottaLuis Villalobos-GallegosAlessandro PosaLuisa AgnelloAlessandro RizzoLuis-Javier HerreraAlessandro RodolicoLuiz Carlos Moraes FrançaAlessandro RossiLuiza Marek-JózefowiczAlessandro ScalaLuka FlegarAlessio ContiLuka PirkerAlessio PaladiniLukas D. LandeggerAlex RamirezLukas FiedlerAlex Siu Wing ChanLukas RasulićAlexander Batista DuharteLukáš ŠkoloudíkAlexander BerezinŁukasz A. PoniatowskiAlexander ChernŁukasz KuźmaAlexander Dimitrov ShinkovLukasz LapajAlexander DityatevŁukasz MałekAlexander GombertŁukasz NawackiAlexander HemprichŁukasz PietrzykowskiAlexander NeefŁukasz RzepińskiAlexander PodgorsakŁukasz SzarpakAlexander RitterLuke ChongAlexander SboevLung-Chi LeeAlexander ScheiterLuo MingyaoAlexander Yu MeigalLusiana Retno AriadiAlexandr CeasovschihLut TamamAlexandra TrivliLütfü AşkınAlexandre Gonzalez-RodriguezLy NguyenAlexandre SzaboLyubov E. SalnikovaAlexandrina S. NikovaM. Carmen Terol CanteroAlexandros G. SykarasM. S. EliwaAlexandros RovasMaciaszekAlexandru BejinariuMaciej GawęckiAlexandru Gabriel BejinariuMaciej Iek SkrzypczakAlexandru VlasaMaciej JaneczekAlexandru-Cristian TucaMaciej JarzebskiAlexey RakovMaciej JedlinskiAlexey SarapultsevMaciej MisiolekAlexis StamatikosMaciej NastajAlfonso Alvarado-LorenzoMaciej RadekAlfonso IbañezMadalina Irina MitranAlfonso Maria PonsiglioneMadalina-Gabriela IliescuAlfredo Malo-MansoMadankumar GhatgeAli AhmadMadhavi TripathiAli CaykoyluMads ThomassenAli JazirehiMael LeverAli Mohammad AlqudahMagdalena A. BryndzaAli Salehzadeh-YazdiMagdalena Da̧browska-GalasAliakbar AlizadehMagdalena DudekAlice BonomiMagdalena IorgaAlice GiontellaMagdalena Mackiewicz-MilewskaAlice GrossiMagdalena OrzechowskaAlice Verticchio VercellinMagdalena RudzinskaAlina Daciana ElecMagdalena ZawadkaAlireza GhoreifiMaged HassanAlireza SouriMaged T. El-GhannamAliseri GovardhanMagesh MuthuAlison EastmanMaha AtoutAlla KushnirMaha Saber-AyadAlvan Emeka Kelechi UkachukwuMahadevan SeetharamanÁlvaro Astasio-PicadoMahdi KhosravyAlvaro DowlingMahendran ChinnappanAlvaro Gallego-MartinezMahmoud AbulmeatyAlvaro García Del CastilloMahmoud ElbattahÁlvaro Monteiro PerazzoMahmoudreza HadjighassemÁlvaro SobrinhoMahmud HasanAlvaro YogiMahmut Cerkez ErgorenAlysia CoxMahnaz KhatibanAlyssa HuffMai Salah El Din Mohamed HelmyAmadeus DobrescuMainak DuttaAmal M. El-NaggarMairi PucciAmanda EltonMaite CaluchoAmanda L. ElchynskiMaja MatulićAmarish YadavMaja PakižAmarnath ChallapalliMaja Rogić VidakovićAmbar Lopez-MacayMajed AlamriAmbreen AzharMajid Najafi KalyaniAmelia FilippelliMakoto FurugenAmelia PietropaoloMakoto TsujitaAmélie DelaporteMałgorzata BlatkiewiczAmelie Isabell StritzkeMałgorzata ChlabiczAmine KhaldiMałgorzata GałeckaAmir Amiri-YektaMałgorzata GrzankaAmir EmamifarMałgorzata JarończykAmir ShemiraniMałgorzata Kęsik-BrodackaAmir SohrabiMałgorzata MarćAmirhossein MirhashemiMałgorzata Polz-DacewiczAmirmasoud AhmadiMaliheh MoradzadehAmit KumarMalik AydinAmit RoshanManal FawzyAmit SinghManas KotepuiAmmar A. OglatManee M. ManeeAmmar Abdullkader BoudakaManickam AshokkumarAmra AdroviçManish CharanAna Álvarez MuelasManisha DixitAna Aurora Díaz-GavelaManit NuinoonAna Carolina OttavianiManohar KodavatiAna ClaveríaManohar MishraAna FaustinoManon NayracAna FigueirasManop PithukpakornAna Florencia Vega-BenedettiMansour HaddadAna I. Álvarez-MercadoMansoureh TatariAna M. Cebrián-CuencaManuel Durán-PovedaAna María Salinas-MartínezManuel GranellAna Moreno-ÁlvarezManuel MuroAna RochaManuel Teixeira VeríssimoAna Rute CostaManuela Adelinde AschauerAna Teresa Corte-RealManuela ChiavariniAna Teresa GabrielManuela ColosimoAna Vujaklija BrajkovićManuela PrioloAnabela Santo Ramalho VenâncioManuele ManciniAnamaria JurcauManvi GoelAna-maria PelinMāra PilmaneAnand Prakash SinghMarc AuriacombeAnanth S. EleswarapuMarc FischlerAnastasios S. TsarouchasMarc Moritz BergerAnbazhagan SathiyaseelanMarcel Rolf PfeiferAnca BojanMarcello MaggioliniAnca Oana DoceaMarcello MiglioreAnca ZguraMarcelo PalinkasAndaleb KholmukhamedovMarcia ManterolaAnders Rosendal KorshoejMarcin FeldoAndrás MihályMarcin PrzybylskiAndrás RabMarcin StraburzynskiAndraẑ DovnikMarcin StraczkiewiczAndré FerreiraMarcin SzerszeńAndre Luiz Pasqua TavaresMarcin ZarowskiAndré M. NicolaMarcin ZielińśkiAndre WannemuellerMárcio De Carvalho FormigaAndrea AngeliniMarco A. ReynaAndrea BallatoreMarco BoAndrea DekanicMarco C. CavacoAndrea DoniMarco CicconeAndrea GeorgiouMarco CordaniAndrea GianniniMarco FoganteAndrea Giuseppe MaugeriMarco JT VerstegenAndrea Gonzalez-MontoroMarco LollobrigidaAndrea GrilloMarco ManfrediniAndrea LatiniMarco NisiAndrea LazzeriniMarco PaciottiAndrea ManciniMarco PalumboAndrea Milostić-SrbMarco PassiatoreAndrea PhillipouMarco PiccirelliAndrea PintorMarco RoscignoAndrea PozziMarco SapienzaAndrea PuppoMarco SchiavoneAndrea R. TeufelbergerMarco ValvanoAndrea RossiMarco ZamoraAndrea SistiMarco ZeppieriAndrea SpiniMarcos Fernando Xisto Braga CavalcantiAndrea Vukić DugacMarcus CoratAndreadi AikateriniMarcus LancéAndreas GatherMarek PaulAndreas MitsisMarek PetrášAndreas NeffMargarita A. SazonovaAndreas RiesMargherita SerraAndreas S. PapazoglouMargot Gage WitvlietAndreea AndronesiMaria A. RadanovaAndreea AvasiloaieiMaria Angeles RojoAndreea Iulia SocaciuMaria Angelica GuimarãesAndreea KuiMaria ArioliAndrei BraesterMaria ArmaouAndrei Marian FeierMaria BabakAndrej ThurzoMaría Carrillo-DíazAndreja FigurekMaría Dolores López FrancoAndrés Alonso Agudelo-SuárezMaria E. GrigoriouAndrés Godoy-CumillafMaría Eulàlia Fernández-MontolíAndrew J. KobetsMaría Felipa SorianoAndrew J. KolarikMaria FortofoiuAndrew James WoodMaria Francisca Colella-SantosAndrew Li YimMaria Francisca CoutinhoAndrew Steven CampMaria Gabriella MelchiorreAndrew W. MaksymiukMaria Gomes Da SilvaAndrey KolobovMaría Granados SantiagoAndrey S. LopatinMaria Helena SantosAndrey V. DubovoyMaria Isabel Hernández-AlvarezAndrija MatetićMaria Jesús Caurcel CaraAndro KosecMaría José Molina-GarridoAndroulla EleftheriouMaria Luiza Anjos PontualAndrzej GlowniakMaria MycielskaAndrzej HeckerMaria NagaiAndrzej SilczukMaría Navarro-GranadosAndrzej TeisseyreMaria OrdonezAndrzej Za̧bekMaria PapaioannouAnestis KarakatsanisMaria PassanteAneta Maria BorkowskaMaria PellicciariAngel CataldiMaria RasoÁngel MonteroMaria Rosa Suñer SolerAngel Romero ColladoMaría Salud JiménezAngela C. GarinisMaria SarapultsevaÁngela María TobónMaria SollerAngela SparagoMaria Teresa GiglioAngélica Saraí Jiménez-OsorioMaria Teresa InzitariAngelika BorkowetzMaria TotanAngelika ChroniMaria UkhanovaAngelika IllgMaria VanoreAngeliki AngelidiMaría-Carmen Sánchez GonzálezAngelo MaffeiMariaevelina AlfieriAngelo NaselliMariagrazia RossiAngelo ScuteriMariamena ArbitrioAngelo TorrenteMariana Avila VeraAnggoro HartopoMariana FloriaAnh Tuan MaiMariana NucciAnida Maria BabtanMariana PalageAniruddha DasMariana R. BottonAnish MukherjeeMariángeles DíazAnita Cybulska-KlosowiczMariann HarangiAnita DeakMarianna E. WeenerAnita Lyssek-BorońMaribel Forero-CastroAnja KühnelMarica MeroniAnja WeiseMarie E. BecknerAnjul KhadriaMarie Saghaeian JaziAnkita PunethaMarie-Bénédicte RougierAnna Andreevna StarshinovaMariela CorralesAnna BersanoMariia D. IvanovaAnna Bogusława Pilewska-KozakMarija HabijanAnna CybulskaMarija JevticAnna CzarnaMarija M. BožićAnna CzopekMarija VukojaAnna Giulia NappiMarika MokouAnna HeffronMarina Gálvez-PeraltaAnna HryniewiczMarina PeballAnna Kasielska-TrojanMarina PiscopoAnna Kołodziej-ZaleskaMario AbateAnna Kutkowska-KaźmierczakMario Cano-MuñozAnna Lucia TorneselloMario DioguardiAnna M. RossMario FruschelliAnna MajdaMario Lozano-LozanoAnna MosiołekMario Mendoza-SagaonAnna Olasińska-WiśniewskaMariola HerbetAnna OlczakMariola JaniszewskaAnna Paradowska-StolarzMariona TerradasAnna PieleszMarios AntonakakisAnna PocurullMarios ConstantinouAnna RatuszniakMarios G. KrokidisAnna RogalskaMarios SpanakisAnna Serra RubertMarisa PapalucaAnna Szumera-CieckiewiczMarius ColinAnna Tyrańska-FobkeMarius CrainaAnna Waśniewska-WłodarczykMarius Lucian CrainaAnna Zalewska-JanowskaMarius MiglinasAnna Ziomkiewicz-WicharyMarius MüllerAnnalisa MaraoneMarius NedelcuAnne Harduin-LepersMariusz NiemczykAnne-Laure RouxMariusz SikoraAnnie D. NiehausMariya A. SmetaninaAnnika Helgadóttir DavidsenMarjan DrukkerAnn-Joy ChengMarjan MohammadiAnoop KumarMarjolein M. EnsinckAnroop NairMarjorie JonesAnshul TiwariMárk AntalAnthony MagitMark D. EwaltAnthony YiiMark D. TricklebankAntoine ChatrenetMark Rami BarakatAntoine NaemMark S. WhiteleyAnton KiselevMarko BaralicAnton MoritzMarko KumrićAntonella BodiniMarkus StorvikAntonella CaminatiMarkus VeltenAntonella ManniniMarkus WettsteinAntonello PennaMarleide Da Mota GomesAntonia KondouMarlena GodlewskaAntonina ShumakovaMarlid Cruz-RamosAntonino Lo GiudiceMarta AltieriAntonino MignanoMarta CampagnoloAntonino NaroMarta ChecchiAntonio BenedettiMarta FijałkowskaAntonio BiondiMarta García-ClementeAntonio Carlos Silva-FilhoMarta GromichoAntonio Eduardo ZeratiMarta LovinoAntonio FacciorussoMarta Martinez AlonsoAntonio GalanteMarta MonzónAntonio Giovanni SolimandoMarta Rojas-GimenezAntonio Gonzalez-BulnesMarta SzachniukAntonio MassaroMartha Alejandra Morales-SánchezAntonio RaffoneMartha Patricia Gallegos-ArreolaAntonio Ramos-MartinezMartijn HoesAntónio Sérgio SilvaMartin Bjørn StausholmAntonio TufanoMartin FaehlingAntonio Victor Campos CoelhoMartin G. SchermAntonio VinuelaMartin JohanssonAntonis SakellariosMartin Jozef PéčAntony PorcinoMartin K. ThomsenAnttonio GiordanoMartin MüllerAnudeepa SharmaMartin Prince AlphonseAnupam MukherjeeMartin RegensburgerApostolos I. TsolakisMartin TeraaApostolos PapachristosMartina FerrilloAraceli Alonso-CanovasMartina IrionAravindhan VeerapandiyanMartina MihaljArbaz SajjadMartina PavlíkováArcady MarkelMartine BaronsArcangelo SebastianelliMartine CrosetArchya DasguptaMartino PavoneArda IsikMarwan GhochArduino ArduiniMary Ann JarvisAriana NelsonMary V. SeemanAriel KorenMarzena LaskowskaAriel Soares TelesMarzena OlesinskaArinjita BhattacharyyaMarzieh SalimiAristeidis KonstantinidisMarziliano NicolaArkom ChaiwongkotMasahiro SugimotoArman GharipourMasahito FushimiArmand FaganelMasamitsu MaekawaArmando CaseiroMasanori MasuiArmon TamateaMasanori MurakamiArnaldo StanzioneMasanori NakataArnaud Didier GermainMasaru TanakaÁrpád TósakiMasashi IzumiyaArpan PatelMasashi YamashitaArpita RaiMasaud ShahArthur De Sá FerreiraMasayuki FujiwaraArtur LemińskiMassimiliano CretaArtur RebeloMassimo CarossaArtur SłomkaMassimo ChiariArturo OrlacchioMassimo LibraArun Kumar RayMassimo RomaniArun Saravanakumar AnnamalaiMassimo TorreggianiArun SwaminathanMassimo TusconiArvin Haj-mirzaianMatej StuhecArya AminorroayaMateusz GrajekArzu Denizbasi AltinokMateusz RadwańskiAshish NoronhaMateusz WiniarczykAshley K. PutmanMateusz ZawadkaAshok IyaswamyMathews VargheseAshok Kumar YadavMathias C. BrandtAshraf KariminikMathieu Di MiceliAshuin Kammar-GarcíaMatias Eduardo Diaz CrescitelliAsker KhapchaevMatias IglickiAskin GulsenMatteo Alessio ChiappediAstgik PetrosyanMatteo FoschiAstrida VelenaMatteo FrigerioAsuman GedikbasiMatteo PaciniAta MahmoodpoorMatteo RipaAthanasia PapazafiropoulouMatteo TrimarchiAthanasios K. KonstantinidisMatteo TurettaAthanasios KiourtisMatteo VattaAtsushi IkedaMatthes HaraldAtsushi SuzukiMatthew B. LanktreeAttayeb MohsenMatthew KoehlerAttila RokaMatthew LarocqueAudic YannMatthew Steven DupreyAudrey AdjiMatthias D. HoferAudrey LafrenayeMatthias GüntherAugusto Garcia-AgundezMatthias UnterhuberAura GabrielMatti HuotariAurel NireșteanMau-Ern PohAurel Popa-WagnerMaulik VyasAurelien LouvrierMaura M. E. PilottiAurelija RadzevičienėMaurice Van KeulenAvinash PremrajMaurilio D’AngeloAxel JakuscheitMaurílio S. De Souza CazarimAxel SchlagenhaufMaurizio Nicola D’AlterioAyako MatsudaMaurizio RomanoAyelén ToroMauro BorzioAyhan KanatMauro MastropasquaAyhan Yahya AcarMauro MonforteAyoung JeongMauro PittirutiAyse SuleymanMax Carlos Ramírez SotoAyslan Castro BrantMaxim SinitskyAzger DusthackeerMaxime BillotAzhar ImranMaxime PichonBahar AtaeiniaMaximilian DietrichBahattin Kerem AydinMaximilian HammerBaicus AndaMay Phu PaingBaki HekimoğluMayank ChoubeyBalamurali Krishna VasamsettiMayank Kumar SinghBalik DzhambazovMayuko Ito FukunagaBálint TamaskovicsMayumi KomineBanu KaraMayumi NishiBanu SalepciMayura MeerangBanzeer Ahsan AbbasiMazhar ZoubiBaptiste LamarthéeMbarek MarwanBarbara Eleni RosatoMd Abdus SubhanBarbara JasiewiczMd Islam SharifulBarbara PirasMd Mamunur RahamanBarbara Ruszkowska-CiastekMd Monirul IslamBarbara SaltzmanMd Rabiul IslamBarbara TorselloMd. Abul Hassan SameeBarbara VodicskaMd. RahmanBaris BengurMd. Tanvir RahmanBartłomiej Henryk NoszczykMeenakshi AhluwaliaBartosz KrzowskiMegan L. GillBartosz RymuzaMeghan MoranBatuk D. DiyoraMehdi MoghaddasiBayodé Roméo AdégbitèMehdi MontazerBeáta HubkováMehmet Ali AltayBeata Labuz-RoszakMehmet Engin TezcanBeatrice GalloMehmet Fatih ŞentürkBéatrice TurcqMehraj AhmadBeatriz MerinoMehran GhaemiBeatriz Solano-MendozaMei-Hwa LeeBeatriz SomozaMeike KählerBee K. TanMeir M. BarakBehrooz JohariMeiyan WangBehrouz ShamsaeIMelanie CareBehzad NarouieMelinda KolcsárBei ChengMelis PalamarBela BalintMelissa RavenBelal ShohayebMellar P. DavisBelen Alonso OrtizMengchen YinBelén Vega-MárquezMenglong XuBelgin AkınMengxi ShenBen MulhearnMenicanti LorenzoBenedict FrancisMerica Glavina-DurdovBénédicte CaronMerlin AriefdjohanBénédicte M. BatrancourtMerry Lynn Noelle McDonaldBeniamin Oskar GrabarekMert İlker HayıroğluBenjamin C. P. LeeMi Kyung ParkBenjamin E. GarfieldMichael A. ProschanBenjamin J. FrischMichael BadowskiBenjamin Q. DuongMichael Boelstoft HolteBenoit DriesschaertMichael D. HellmanBenoit MichotMichael D. SpartalisBeom Kyung KimMichael DevaneBernard SpeculandMichael F. BeilBernd W. SiguschMichael FishmanBernhard GibbsMichael FroschBernward LauerMichael KaabakBert VandenberkMichael KnitschkeBertalan MeskóMichael LichtenauerBertil RydenhagMichael McDowellBeth Mozolic-StauntonMichael NekludovBettina BlaumeiserMichael RehmanBhakti BasuMichael RoseBhanwar Lal PuniyaMichael S. JacksonBharat GurnaniMichael SchörghuberBharath Kumar VelMichael SchwakeBhaskara Rao ChintadaMichael T. KoltzBhavani S. KowtharapuMichael TomassonBhaven ModhaMichael WiblishauserBhavin VasavadaMichail DiakosavvasBi ZhaoMichail KlontzasBiagio SantellaMichail Zografakis SfakianakisBiagio SolarinoMichal AlexovičBibhuti Bhusan DasMichał BorysBidisha RoyMichal KorostynskiBiliang ChenMichal NowickiBinbin SunMichal SarulBingyang JiMichal WozniakBinu Kandathilparambil SasiMichał ZarobkiewiczBiswajit PadhyMichele D’AttilioBita GeramizadehMichele Di CosolaBlazen MarijicMichele FinottiBo HuMichele GallaminiBo WangMichele GolinoBobak MosadeghMichele MagnocavalloBonan ChenMichele MarchioniBoris Filipović-GrčićMichele PietragallaBożena Szyguła-JurkiewiczMichele TorreBrandon Peter Lucke-WoldMichel-Edwar MickaelBranislav KollarMickael EssoumaBrega CarlottaMiguel A. OrtegaBrian BellMiguel Ángel Castro VillamorBrian BieberMiguel Ángel Román-RodríguezBrian CicaliMiguel Castelo-BrancoBrian K. FoutchMiguel Castelo-Branco SousaBrian T. DavidMiguel Urina-TrianaBrigida BochicchioMihaela Flavia AvramBrígida Ribeiro PinhoMihaela HedeșiuBrocha SternMihaela MocanBruce I. RapleyMihaela PerteaBruna PanizzuttiMihaela VladBruna Santos Da SilvaMihaela-Adela VinţanBruno FiondaMihai Dorin VartolomeiBruno FossoMihai MusteataBruno MilletMihnea-Alexandru GămanBruno Pereira CrulhasMikaela PolingBruno RegoMikhail KhvostovBryan MathisMikhail KislinBülent ErginMikhail M. KostikBurcin CelikMikhail PyatnitskiyByeong-Wook SongMikhila MahendraByoung-Eun YangMiki KushimaC. James SungMikiko TokiyaCai-Mei ZhengMikio OkazakiCǎlin HomorodeanMikolaj DabrowskiCalin TataruMikyoung ParkCamila TirapelliMilan LepicCamilla PredaMilen DimitrovCamilla RussoMilena Adina ManCamille SchwabMilica BorovcaninCan Fahrettin KoyuncuMilica DeklevaCandelaria Martín-GonzálezMilos MatkovicCarina De Fátima RodriguesMin Kyu KimCarl BeckerMin Young LeeCarl J. LavieMine BekarCarl MannMine Gürsaç ÇelikCarlo CaiatiMine Kanat-PektasCarlo CernettiMinehiko InomataCarlo CinqueMing Hsien TsaiCarlo PietrasantaMing YangCarlo ZaninettiMingchong YangCarlos A. M. CarvalhoMingkai ChenCarlos Delgado-MiguelMing-Ping WuCarlos Díaz-CastilloMinh Hoang NguyenCarlos DurántezMin-Ho HanCarlos Llanes-ÁlvarezMinoru GotohCarlos MestresMiodrag FilipovicCarlos Navarro-CuéllarMircea ChiricaCarlos O’Connor-ReinaMircea TampaCarlos UbedaMireille L. A. Van GoethemCarlotte KiekensMirela TiglisCarl-Philipp JansenMiren AltunaCarmela PaolilloMiri KrupkinCarmelina RuggieroMiriam Geal-DorCarmelo CorsaroMiriam Saiz-RodrigezCarmen Dora Méndez-HernándezMirian Santamaría-PeláezCarmen Méndez HernándezMirjam SchuchardtCarmen OrtizMirjana TurkaljCarmine IzzoMirko ManchiaCarol Yen Chin LinMirna FawazCarolina GiordanoMiro JukićCarolina MaioraniMiroslav IlićCarolina Oliveira De LimaMiroslav P. IlićCarolina RomeroMiroslava NedyalkovaCarolina SimioniMirosław AndrusiewiczCarolina Susana CerrudoMirto FolettoCaroline RaynalMisbahuddin M. RafeeqCaroline SubraMishra Vikash ChandraCarrie R. HowellMislav MikusCarsten HoffmannMiso GostimirCatalin BulaiMithra Nidharsh HegdeCatalin ZahariaMithun RudrapalCatalina MihaiMitra AkbariCaterina DaporMitsushige SugimotoCaterina MancarellaMi-yeon EunCathalijn LeenaarsMoamen Salah S. RefatCatherine DejeanMobeen RehmanCatherine E. CreeleyMohamed A. R. SolimanCatherine M. SteinMohamed AliCátia DominguesMohamed AttiaCatriona KellyMohamed HassanCecilia BoveMohamed M. Abdel-DaimCelia GarauMohamed MediouniCelina-Silvia StafieMohamed MohanyCeres Fernandez-RozadillaMohamed SallaCesare C. BerraMohammad A. SaadChahnez Charfi TrikiMohammad Afzal KhanChahrazade El AmriMohammad AlamChaitanya P. PuranikMohammad Al-WardatChamara V. SenaratnaMohammad ArjmandChamindie K. PunyadeeraMohammad AshfaqChander Shekhar MukhopadhayayMohammad EtoomChandika GamageMohammad Fahad UllahChandrashekar JanakiramMohammad KhisheChangcai BaiMohammad Mahbubur Rahman Khan MamunChangchen ZhaoMohammad Reza Khodaei ArdakaniChanpreet SinghMohammad Safiqul IslamChan-Yen KuoMohammad ZulkifliChaofan LiMohammadreza ShalbafanChariklia Tziraki-SegalMohammed Abou RehabCharis NtakoliaMohammed E. ChoudhuryCharles NortonMohammed Oday EzzatCharles W. LanksMohammed S. ElSheemyCharlie WykoffMohammed YosriCharlotte Toftmann HansenMohammed ZawiahCharny ParkMohsen AminizadehChen Liang TsaiMohsen Mosayebi-SamaniChen YangMoito IijimaCheng WangMojca LunderChengfei JiangMojgan GharipourChengwei HuangMojtaba MokariChenhan ZhongMojtaba VaismoradiChenyu LiMomcilo JankovicCheong Hoon SeoMona VintilaCheuk Lun LiuMonia GarofoloChewTeng KorMonia MarchettiCheyuan KuoMonica BassoChhagan BihariMonica CurròChia-Hong YengMónica MartinsChiaki ToidaMónica Muñoz-ÚbedaChian Ju JongMonica Steluta MarcChiao-Yin SunMonika GjorgjievaChiara BellocchiMonika KujdowiczChiara ChieregoMonika PrakashChiara De-ColleMonjoy SahaChiara Di RestaMontserrat SantamariaChiara PaoliniMorgan BroggiChiara PiliegoMorgan D. SalmonChiara RomaniMoritz C. DemlChiara RomeiMorteza Fallah KarkanChiara SorberaMostafa BijaniChia-Yu ChenMotohiro MunakataChi-Chih HungMuh Imam AmmarullahChi-Chun HoMuhammad A. JawadChieh-Lin TengMuhammad Abdullah KamranChien-Chang LiaoMuhammad FarooqChien-Li ChenMuhammad IrfanChih-Chi ChenMuhammad M. FurqanChi-Horng LiaoMuhammad N. ZahidChih-Po ChiangMuhammad RiazChih-Yang WangMuhammad Shahid Riaz RajokaChin Hui SuMuhammad SyafrudinChina NaganoMuhammed Sedat SakatChing-Nung WuMuharrem AkinChinmay Kumar PandaMukesh Kumar SriwastvaChinna Venkata Subbaiah KadiamMukesh VermaChiranji Lal ChowdharyMulazim Hussain AsimChisato KinoshitaMunawar NaylaChong-Chi ChiuMurali MamidiCho-Yi ChenMurat AladaǧChris BusbyMuriel ElhaiChristelle CorauxMuriel Ramírez-SantanaChristian A. MuellerMurugabaskar BalanChristian A. PogodaMustafa AkkayaChristian BeltzerMustafa Ozan HorsanaliChristian BleilevensMuthu SathishChristian HöhneMyrto PapamentzelopoulouChristian KlimekMyung Goo LeeChristian LiebschNada BinmadiChristian Mata MiquelNadeem BilaniChristian NapoliNadezhda G. GumanovaChristian SetzNadia M. HamdyChristian SmolleNadia Maria SposiChristian WöberNadine HornigChristian ZanzaNadinne RomanChristina Anna StratopoulouNaeem JanChristina KrantzNaga Babu ChinnamChristina LaukaitisNagwa Ali SabriChristine E. Schaner TooleyNagwan SalehChristine Marie MillsNaim Abu FrehaChristine TedijantoNaina Bhatia-DeyChristof RöösliNaita Maren WirsikChristoph FaschingerNalini DhingraChristoph JochumNam HuynhChristophe BattailNamita JayaprakashChristopher BoehlkeNan JiangChristopher HillNan YangChristopher J. WinfreeNanako KawaguchiChristopher NimskyNancy Monroy-JaramilloChristopher V. HughesNannan LuChristos FeloukidisNaoki YamamotoChristos V. RizosNaoro TanakaChristos-Konstantinos AntoniouNaoyuki MorishigeChun LiangNaoyuki TakahataChun Nam LokNarayanaganesh BalasubramanianChundi ZhengNardos AbebeChung-Hui LinNarin PetrotChung-Ming ChenNaseer AhmedChung-Ying LinNaser AlsharairiChun-Ming ChenNastaran EmaminejadChun-Wu TungNatalia A. BezdenezhnykhChunxu GaoNatalia Albaladejo-BlázquezChunyan ChengNatalia FilippovaChyong-Huey LaiNatalia GrubaCihan BedelNatalia StepanovaCintia E. CitterioNatalia V. YunusovaCinzia CasuNatalie Desiree KlassCiprian N. SilaghiNatalie DeuitchCiro Pasquale RomanoNatalija VedmedovskaClaar Debora Van Der Maarel-WierinkNatasa PopovicClaes FrostellNathalia PadillaClaire De BisschopNathalie Van Den BergeClaire-Marie RangonNathan Martin D’CunhaClara GreenNathan SeligsonClara NerviNathaniel H. RobinClare McKeaveneyNatsuo TomitaClarence KhooNaushad Ali JungleeClaudia CrimiNava ZisapelClaudia LermaNavid Jubaer AyonClaudia LoretiNavid SobhaniClaudia MartiniNazario FoschiClaudia MehedintuNazefah Abdul HamidClaudia PisanuNeamtu BogdanClaudia Reyes-GoyaNebojša D. ZdravkovićClaudia Talero-GutiérrezNebojsa S. ManojlovicClaudia Tovar-PalacioNeda MostafaeeClaudio LuchinatNektaria PalaiologouClaudiu MargineanNelson RodriguesClinton WebbNelson TangColin M. SmithNémeth BalázsColleen AldousNenad BukvicConcepción De Hita CantalejoNeriman ÖzkanConsolato M. SergiNessy JohnConstance C. F. M. J. BaatenNevo MargalitConstantin T. LucaNguyen Minh DucConstantino BalestraNhu NguyenConstantinos PitsiosNianhui CuiConsuelo Pedrón-GinerNicholas F. TaylorCorentin J. GoslingNicholas J. AndersenCorina BocsanNicholas LeeCorina VasileNick KantaputraCornelia AmalineiNick SpindlerCorrado AngeliniNicola CavasinCorrado CiattiNicola FerraraCorrado TagliatiNicola MarottaCostas S. ConstantinouNicola MondanelliCristian DeanaNicola MontanoCristian ScheauNicola MontemurroCristian TrambitasNicola OrzalesiCristiana Iulia DumitrescuNicola VanacoreCristiana VidaliNicola ZeniCristiano FavaNicolae Dan TesloianuCristina AurigemmaNicolae GicaCristina CarbonellNicolae-Florin CofaruCristina CavinatoNicolas FarinaCristina Crenguţa AlbuNicolas GiraudeauCristina García-BravoNicole BechmannCristina GomezNicole DempsterCristina GrippaudoNicoletta BigliaCristina Iulia MitranNicolette V. RomanCristina TiuNicolò SellaCristina TudoranNicolo ZarottiCristoforo PomaraNicos MaglaverasCristopher Van HoutNiels QvistCrystell Guadalupe Guzmán PriegoNigel FarrowCyril FersingNihad AdnanCyrus MotamedNikhil AgrawalD. Gareth EvansNikhil JaganDahang YuNikhlesh SinghDahui WangNikola PavlovićDaichi SoneNikola StefanovićDaiji NagayamaNikolaos A. ChrysanthakopoulosDaisuke NishizawaNikolaos AntonakopoulosDalia MedhatNikolaos GkantidisDamian KaniowskiNikolaos KathopoulisDan ChenNikolaos KoukouliasDan Cristian RaduNikolaos MachairasDan HabermanNikolaos PistamaltzianDan Van BuiNikolaos SchizasDana Carmen ZahaNikolaos TsetsosDana Farengo ClarkNikolay A. BarashkovDana L. AultNikolay BugaevDandan WuNikos ViazisDaniel Asunción-AlvarezNilanjana BanerjeeDaniel BoegerNimrat ChatterjeeDaniel C. MorrisNina HallowellDaniel CruzNina Isabel Mendez DominguezDániel CzurigaNina MendezDaniel García IglesiasNina RembiaŁkowskaDaniel Gonçalves-CarneiroNina SperberDaniel HackettNina TyutyushevaDaniel HagenfeldNingxi YangDaniel HeiseNiroj Kumar SahooDaniel HierNisansala AbeyrathnaDaniel L. PiskorzNiteshkumar AgarwalDaniel LeucutaNithesh NaikDaniel LiedtkeNiya MilevaDaniel LighezanNizamettin GüzelDaniel López-LópezNoelia María Martín EspinosaDaniel Octavian CostacheNoemi CoppolaDaniel P. PotaczekNoha Mousaad ElemamDaniel P. ZalewskiNoor Nabilah Talik SisinDaniel PecsiNoppawan MoralesDaniel Peña-OyarzúnNoriane Andrina SieviDaniel Reis WaisbergNorihiko NaritaDaniel S. PrattNorio ImaiDaniel San-JuanNorio YamamotoDaniela CaccamoNoriyasu HirasawaDaniela CalinaNorm O’RourkeDaniela CavacoNoura AlsufyaniDaniele ArmocidaNuri Baris HasbalDaniele CastellaniNúria FarréDaniele CorboNuryani NuryaniDaniele GianfrilliOana AlmasanDaniele GiansantiOana Cella AndreiDaniele MasaroneOana Cristina ArghirDaniele UrsoObara KojiDanijela PiskulicOctav Marius RussuDanijela TasicOctavian AndronicDanuta NowickaOdette ViñasDanyi LuOlaf EberhardtDariga AkashevaOlan Jackson-WeaverDarinka KorovljevOlarik SurintaDario BrambillaOleksandr SverdlovDarío GarigliottiOlga AnatskayaDario LeoneOlga BalafaDario MessenioOlga Di FedeDarius J. UnwalaOlga Maria Pimenta Lopes RibeiroDariush IraniOlga OzolineDarlene MitranoOlga ShchaginaDarren LauOlga SysoevaDaryl NiedermoserOliver J. WatkeysDavid A. RizzieriOliver KalpakDavid Alan RizzieriOliver R. SegalDavid EdholmOliver RiesenbeckDavid FoxeOliver SommerfeldDavid GreenhalghOlivera Mitrović AjtićDavid HeberOlivier SterkersDavid JesenkoOluwatosin OluwadareDavid KeithOluwatoyin A. AdelekeDavid LavilleOmar El HibaDavid MolnarOmar Enzo SantangeloDavid OharaOmar Farookh PinjariDavid Ramiro-CortijoOmar HamdyDavid ReillyOmar M. ShamiehDavid Ribas PérezOmar MustafaDavid T. LiuÖmer Faruk AkçaDavid WeikOmran SaifiDavid ZweikerOnofrio LaselvaDavide CarusoOrestis GiotakosDavide CitterioOrsolya BiroDavide DonnerOsama Y. SafdarDavood KhaliliOsamah AlwalidDawid MadejOsamu ImatakiDawid StormanOsamu IshidaDeasy IrawatiOskars KalejsDebabrata ChowdhuryOsvaldo D. MessinaDebojit BoseOurlad Alzeus TantengcoDebopam GhoshOv Daniel SlaydenDeborah VerranOvidio De FilippDebra BoeldtOvidiu SirbuDech DokpuangOwen Martin WilliamsDeepa KrishnaswamyÖzcan YavaşiDeepak Kumar KhajuriaÖzüm Yuksel TunçyürekDeepika KumarP. JideshDeepti ShrivastavaPaloma Malagon LopezDemin CaiPanagiota XaplanteriDeng-Ho YangPanagiotis I. GeorgianosDenis MiyashiroPanagiotis KatrakazasDeniz Can GuvenPanagiotis MourmourisDeniz EkizPanagiotis N. TsikourasDennis AdjepongPanagiotis TsagozisDennis MeestersPanayiota PetrouDesmond AgboadaPanda SantoshDespina FotiouPankaj AhluwaliaDhaval ChauhanPanthier FrédéricDhaval PauPaola BianchiDhiran VerghesePaola FerrariDhiren D. PandyaPaola PierucciDhivya KumarPaola QuaresimaDhruba PathakPaolo AbondioDi Miceli MathieuPaolo Albino FerrariDi WuPaolo BoffanoDiana Cristina Pérez-IbavePaolo BrusiniDiana GiannarelliPaolo CalistriDiana MartinsPaolo CampioniDiana ȚînțPaolo CastellucciDiane M DolezelPaolo Emilio PudduDianjun CaoPaolo MeneguzzoDiederick DuijveszPaolo MonardoDiederik De CockPaolo Riccardo BrustioDiego Fernández LázaroPaolo ScudieriDietmar R. FriesParashar DhakalDimitri KakabadseParaskevi TrivilouDimitrios KasselimisParastoo TarighiDimitrios KotsirisParisis GallosDimitrios N. NikasParul VarmaDimitrios PanagopoulosPascal StammetDimitrios VlachodimitropoulosPascale FanenDimitris LambrouPasquale AmbrosinoDimitris TatsisPasquale AuricchioDimy FluyauPasquale AvellaDinesh MedipallyPasquale DitonnoDiogo Guedes VidalPasquale ParibelloDionysios C. TafiadisPasquale StefanizziDipenkumar ModiPasquale ViggianoDirk BisterPathak Chandramani M.Dirk BöhmerPatil ShankargoudaDirk J. Van LeeuwenPatricia Acosta-VargasDisha MalaniPatrícia Aline Gröhs FerrarezeDisha SharmaPatricia LalorDivya GopinathPatrícia Manarte-MonteiroDivya KandariPatricia SchofieldDivya RadhakrishnanPatricio Más-SerranoDivya SahuPatricio Orellana-PalmaDiwakar Bastihalli TukaramraoPatrick DansetteDmitri NepogodievPatrick DunnDmitri ShchekochikhinPatrick H. J. HemmerDmitrii TumakovPatrick HerrDmitriy IvashchenkoPatrick LüningschrörDmitry S. PanfilovPatrizia LoPrestiDoerte WichmannPatrizia StracciaDoga KuruogluPatrycja KleczkowskaDoina A. TodeaPatrycja S. MatusikDoina AzoicaiPattharawin PattharanitimaDomenico De BerardisPaul Christopher MarcoglieseDomenico FerraioliPaul E. StevensDomenico LioPaul EngeroffDomenico MallamacePaul K. MaciejewskiDomenico Maria PagliaraPaul LacazeDomenico TricaricoPaul LinksDominik GriebPaul M. GarrettDominik OlejniczakPaul Matthew Dimaguila PascoDominik PromnyPaul OakleyDominika ZającPaul RobinsonDonal G. SinexPaul ValleDonald B. GiddonPaula TavaresDonatella GiulianiPaulina AdamskaDone StojanovPaulina CeglaDongdong WangPaulina MisztakDonghwan ParkPaulo BettencourtDongjing LiuPaulo Magno Martins DouradoDongqing WeiPaulo PalmaDongsheng FanPaulo Santos-CostaDorin GheorghePavel P. KuksaDorin IonescuPavel ŽáčekDoris RusicPavle AndjusDorival Mendes Rodrigues JuniorPavol ZelinaDorota WójcikPaweł AbramowiczDoru DiculescuPaweł GaćDragan JeremicPawel KiperDragana BajicPawel KleczynskiDragana LazarevićPaweł KrukowDragos PredescuPayam BehzadiDrew NassalPedro L. MeloDubravko HabekPedro M. MagalhãesDumitru CiolacPedro Manuel Rodríguez-MuñozDuncan James McLauchlanPedro Miguel RodriguesDuraiyah ThangathuraiPedro Silva CunhaDurán MercedesPeggy MandersDusica PahorPei-Chen LiDylan M. JohnsonPei-Chin LinE Scott SillsPei-Li YaoEddy J. DavelaarPekka PorelaEddy KruegerPeng FangEdgar CambazaPengcheng YangEdina PolettoPengfei JiaEdinson Dante Meregildo RodriguezPeng-Hui WangEdith Lillian Roth GjevjonPengwei LuEdoardo BraunerPengzhou HangEdoardo IsnaldiPepper SchedinEdoardo MalfattiPershina OlgaEdoardo Maria MuttilloPetar AvramovskiEdoardo MercadantePetar OtaševićEdoardo MonfriniPeter BaptistaEdouard CoeugnietPeter BentzerEdouard GerbaudPeter CnuddeEdsaul Emilio Perez-GuerreroPeter CrouchEdson Santos Ferreira-FilhoPeter KeoghEduardo Andrés-LeónPeter KokolEduardo BonavitaPeter LuntEduardo DominguezPeter StankoEduardo Rodríguez-NoriegaPetra A. ThürmannEduardo Vieira NetoPhanikanth JogamEdvardas DanilaPhilip L. MarEdward HofferPhilip SarajlicEdwin SeetPhilippe GarrigueEfraín Santiago-RodríguezPhilippe Matthias TschollEfrat Shavit-SteinPhilippe MauryEfstathios ChronopoulosPhilippe OriotEfstratios-Stylianos PyrgelisPhilippe PérotEhsan DowlatiPhilippe R. KoninckxEhsanul Hoque ApuPhilippe TausskyEiichi WatanabePier Luigi AntignaniEirini Kanella PanagiotopoulouPierangela BrunoEirini OrovouPierangela TottaEitaro KodaniPiergiorgio IannoneEkaterina A. TrifonovaPiergiusto VitulliEkaterina A. VeniaminovaPierluigi TosEkaterina SilinaPiero PaladiniEl-Anwar Mohammad WaheedPierre D. MouradEleftherios AnastasopoulosPierre GiguèreElena BernadPierre SimeoneElena Chertok ShachamPietro Andrea BonaffiniElena GubarPietro BertiniElena MarchioriPietro C. DattoloElena Natera-VillalbaPietro CarotenutoElena ObradorPietro MaranoElena OlivettiPietro MazzeoElena PeevaPietro ScicchitanoElena PrietoPilar Garcia IglesiasElena ShakhtshneiderPilar Martín-EscuderoElena V. GerasimovaPing YangEleni KarlaftiPing-Chieh PaoEleni KletsiouPing-Chung LeungEleonora TopinoPingfan SongEleuterio A. Sánchez-RomeroPing-Tao TsengEleuterio SánchezPinnita PrabhasawatElham AhmadianPiotr BiałasElham BidabadiPiotr CzarneckiElham FaghihzadehPiotr DziegielElham MasoumiPiotr H. SkarżyńskiElham RazmpooshPiotr KonopelskiEli M HeifetzPiotr MalaraEli RistevskiPiotr MorasiewiczElia ParadisoPiotr WlaźEliano CascardiPiumi LiyanageElias B. RizkPo-Ching WangElias DritsasPo-hsiang LiaoElias KehagiasPolyxeni MantzouratouElie El HelouPongsakorn TantilipikornElie H. MotulskyPooja SonavaneElisa BaratellaPoonam YadavElisa BelluzziPo-Yu FongElisa ContiPradeep SinghElisa DigoPradipta BhaktaElisa J. F. HouwinkPramod GaurElisa Martín-MontañezPranab DeyElisa RussoPranshu SahgalElisabeth BloemenaPrasad TrivediElisabeth Sornay-RenduPrashanth Kumar Bhusetty NageshElisabetta BertelliniPrashanth ThevkarElisabetta CalettiPraveen Kumar Raj KumarElisabetta PolizziPreston E. BratcherElisha KrasinPrince AtorkeyEliza RussuPritesh P. JainElizabeth SecordPriya DeshpandeElla KimPriya RamanathanEllen HillegassPriya VeluswamyElmsellem HichamPriyanka MishraElroy Patrick WeledjiPriyanka SandalElsa VitaleProttoy HasanElshymaa A. AbdelnabyPrzemysław DudaElvira BratilăPrzemysław KołodziejElżbieta CiporaPrzemysław Robert NowakowskiElżbieta Czekajska-ChehabPushpa RaoElżbieta PoniewierkaQaiser RiazEma VrbanovićQi FangEmad IbrahimQi GaoEmanuela D’AngeloQi JinEmanuele AmodioQian LiEmanuele BarabinoQian YangEmanuele GallinoroQiang ZhuEmanuele MarzettiQichang MeiEmanuele MondaQifei LiEmese MezõsiQimin HaiEmilia JaskułaQin Xiang NgEmilia OgodescuQing MaEmilia SorokoQing PanEmilien JeannotQinghua CaoEmilio PortaccioQingxin MuEmir M. MuzurovićQinqin PuEmma D’IppolitoQisheng YouEmma Kurnat-ThomaQiuliyang YuEmmanouil V. DermitzakisQuang Hung DoEmmanuel GabrielQuan-Hu ShenEmmanuel KontomanolisQuanshun LiEmmanuel Navarro-FloresR. Douglas WilsonEmmanuel Silva MarinhoRabia Sannam KhanEmre BilginRachael E. CliffordEndrit ShahiniRachael HachadorianEng Lee OoiRachel HuddartEngy ElekhnawyRachel KalmannEnikő KovácsRachel Wah Shan ChanEnis Alpin GüneriRachelle MendozaEnrico CheccucciRachid Ait AddiEnrico ContiRachid Tahiri Joutei HassaniEnrico SpinasRachna ShahEnzo Maria VingoloRadmila Capkova FrydrychovaEpameinondas GousopoulosRadmila JankovićErand LlanajRadmila KaranErbil KaramanRadosław GocołErdal KaravasRadoslaw JaworskiErez Bar-HaimRadoslaw Marek KiedrowiczErhan OkuyanRadu BotezatuEric A. EppingRadu C. RacovitaEric ChappelRadu MitrutEric E. VinckRadu OlariuEric HuetRadu T. StoicaEric RytkinRafael Baltierrez-HoyosEric S. HoRafael BernardesErica GentilinRafael Vidal-PerezErich CosmiRafaella Pessoa MoreiraErik FraunbergerRafał GerymskiErik KarlssonRafal KocylowskiErika Bevilaqua RangelRafał RójErkan TopkanRaffaela PeroErnő ZádorRaffaele CampisiErwin BrosensRaffaele SerraEsa JantunenRagab Kamal ElnaggarEsmaeil MohammadnejadRaghavendhar R. KothaEsmeralda JuárezRahbel RahmanEssam A. TawfikRahul K. KherEssam Al-MoraissiRahul KrishnatryEsteban Obrero-GaitánRahul SanawarEsteban SepúlvedaRaimund WinterEstefania NuñezRainer SchmittEstela SalagreRaja ChakrabortyEstelle Ayme-DietrichRajeeb Kumar SahEswar ShankarRajeev GuptaEszter DuczaRajeev SinglaEtongola Papy MbelambelaRajeev TiwariEttore DinotoRajendra Prasad SettemEugène A. A. RameckersRajendran BoseEugene KucharzRajesh Kumar TripathyEugenia ScaricamazzaRajikha RajaEumorfia KondiliRajiv DoddamaniEun Jeong GongRajiv SahEun-jung LeeRakesh Kumar ChandraEusebio ChiefariRalf KetterEustaquio AbayRam RamamurthiEva BagyinszkyRam SagarEva Krieghoff-HenningRam SharmaEva ObermayrRamachandra BarikEva RealiRamachandran PrakasamEvaldo FaviRamanagouda BhojappaEvan S. LeibnerRamces Falfan-ValenciaEvangelia Eirini TsermpiniRamendra Pati PandeyEvangelia KoukakiRami S. KantarEvangelos PantelisRamin JaberiEvelyne Jacqz-AigrainRamzi MahmoudiEvgeny E. BezsonovRaozhou LinEvgeny ErmakovRaphael CiumanEvgeny MikhaylovRaquel Sanabria-De-La-TorreEwa DudzińskaRareş Mircea BîrluţiuEwa Gibuła-TarłowskaRasa MladenovicEwa Jȩdrzejczyk-PatejRasa SabaliauskaitėEwa LangeRasa ZarnegarEwa MichalakRasha MakkiaEwelina DziurkowskaRasheed Omobolaji AlabiFabien KaweckiRasoul GhasemiFabio Matos ChiarelliRaul Cruz-CanoFabio ScarpaRaúl Juárez-VelaFabio ScoppaRavi Kumar SharmaFabio VitaglianiRavi Philip RajkumarFabrizio D’AcapitoRaymond H. KimFaez Saleh Al-HamedRayudika Aprilia Patindra PurbaFahad QuhalRăzvan George RahotăFaisal F. HakeemRăzvan-Ionuț PopescuFaizah Safina BakrinRebecca PanconesiFan-Fen WangRebecca S. EshraghiFang HouRebecca SenettaFangming XieRedha AliFangyao TangRehab Mahmoud Abd El-BakyFang-Ying ChiuReidar P. LystadFanny O. ArcolinoReiki NishimuraFarbod KhameneifarReinhard KoppFares E. M. AliReinhard Schwartz-AlbiezFarhad Soleimanian GharehchopoghReinhold FeldmannFariba Behnia-WillisonReka AlbertFarid SaadRekha JagadapillaiFarideh Jalali-MashayekhiRemo LazazzeraFarnad ImaniRenata Novak KujundžićFarzad Taghizadeh-HesaryRenata PecoticFarzaneh KordbachehRenata VrsalovicFatemeh KhatamiRenata ZadroFatemeh Sanie-JahromiRenata ŽunecFaten El SayedRenato MeloFatima MubarakRenaud GeslainFatmanur Tuǧcu-DemirözRené DaherFazil AlievRené WubbelsFeby SaviraRenee RieneckeFedele LemboRenin ChangFederica BelloneRen-Jun FengFederica BuccinoRenzo MarcolongoFederica Di SpiritoReto BaertschigerFederica DomatiReuben Olugbenga AyelekeFederica GalimbertiReyhan ArslantaşFederica GaniReza RanjbaranFederica Genovesi-EbertReza ShahriariradFederica PianiRhona MacLeodFederica VeroneseRicardo A. Cartes-VelásquezFederico LonghiniRicardo GarciaFederico Manuel GiorgiRicardo LoureiroFederico MeiRicardo Pardillo-DíazFedor LurieRiccardo Antonio RicciutiFedor NovikovRiccardo PozzanFelix A. RuizRichard BolesFelix AdochieiRichard DekhuijzenFeng LiuRichard ParrishFeng YuanRikinkumar S. PatelFerdinando AgrestaRintu ThomasFerdinando FranzoniRishi Man ChughFerdinando MannelloRita FloresFernanda Da Cruz Landim- AlvarengaRita FragosoFernando CardonaRita PavasiniFernando GilsanzRita SeeboeckFernando HernanzRitthideach YorsaengFernando Muñoz-HernándezRitu GuptaFernando Reinaldo RibeiroRobby MarkwartFernando ValenzuelaRobert BaranFerruh ArtuncRobert BłaszczykFeten Fekih-RomdhaneRobert BreitkopfFilip MessRobert C. BahlerFilipe Manuel ClementeRobert ĆelićFilipe PintoRobert DraguFilippo BanchiniRobert LeonardFilippo CademartiriRobert M. HermannFilippo ManelliRobert PowersFilomena De NigrisRobert PrillFilomena NapolitanoRobert S. BenjaminFlavia Fondevila PeñaRobert Shyh-Ming ChenFlavio BertiniRobert TroticFlora LiottiRobert WeinzierlFlora N. BalievaRoberta IaconaFlorent CarsuzaaRoberta RicciarelliFlorian EmmerichRoberto AlberaFlorian Markus ThieringerRoberto Alonso González LezcanoFlorian SchneiderRoberto BiniFlorin George HorhatRoberto MenèFlorin Ioan ElecRoberto PalumbiFlorin MituRoberto PeltriniFortunato IacovelliRoberto Rodríguez-JiménezFouquet GuillemetteRoberto RonaFranca BarbicRoberto ScarpioniFranca FagioliRoberto ScicaliFrancesca Anna CupaioliRoberto VagnettiFrancesca CottiniRobin WilsonFrancesca Lea SaibeneRocio Alejandra Chavez-SantoscoyFrancesca NapolitanoRocío Llamas-RamosFrancesca WirthRoddy HiramFrancesco BennardoRodney HullFrancesco CardaioliRodolfo RedaFrancesco CeiRodolfo Rocha Vieira LeocádioFrancesco ChianconeRodrigo Romero-NavaFrancesco CiodaroRoel Van OvermeireFrancesco CollivignarelliRogelio González-GonzálezFrancesco CoreaRoger E. ThomasFrancesco CucciaRogério Leone BuchaimFrancesco D’AmicoRohit ShuklaFrancesco De FrancescoRohollah NasiriFrancesco Di GennaroRokas RačkauskasFrancesco Di LorenzoRoland MertenFrancesco GiangregorioRolf TeschkeFrancesco GrandeRoman MaslennikovFrancesco MaffìaRoman MichalikFrancesco SalzanoRoman PanovskýFrancesco SommaRoman SmolarczykFrancesco StiloRomina Marina SimaFrancis CucinottaRon ShapiroFrancis Jesmar P. Montalbo MontalboRonald B. BrownFrancisca García-Moreno NisaRonald OeiFrancisco Alcantud MarínRong Stephanie HuangFrancisco CastilloRoni SettonFrancisco GermainRoopa BiswasFrancisco Guzman PrunedaRosa Estrada ReyesFrancisco Javier García-FernándezRosa Malo De MolinaFrancisco Javier Rodríguez-LozanoRosa María Cardenal-PirisFrancisco Navarro-TriviñoRosalba PerroneFrancisco Rodrigues PintoRosana ShuhamaFrançois MargueritteRosanna CardaniFrançois MontagneRose Gubitosi-KlugFrank BraunRoseli NomuraFranklin DexterRosemary CaronFranz Felix KonenRoshan SatangeFrederic BarbeyRottem KuintFrederic DenisRoxana Cristina RimbaşFrederik PetersRoxana Liana LucaciuFredrick EzeRoxane Isabelle KestnerFriederike BachmannRoy RobertsonFriedrich MedlinRubén Arroyo FernándezFrikkie J. W. CalitzRubén Pérez-ElviraFruzsina Molnár-GáborRubina ShaikhFu LuoRuchi BhandariFuad K. HashemRuchi RoyFuhito HojoRudolf KolozsváriFuhong CaiRui HenriqueFumiaki IsohashiRuifeng HuFumiaki ShimizuRumiana TzonevaFumihiro OgawaRuna KuleyGaber PlavcRundk HwaizGábor Nagy-GróczRupert ConradGabriel Dias RodriguesRusan CatarGabriela C. ZaharieRussell D’SouzaGabriela CiavoiRussell F. D’SouzaGabriela De Morais Gouvêa LimaRustem ZairovGabriela Ildiko ZondaRute PereiraGabriela TeneaRuth Dudek-WicherGabriela-Dumitrita StanciuRuxandra Diana SinescuGabriele DonzelliRuxandra-Maria MihaiGabriele GhettiRwik SenGabriele MelegariRymantė GleiznienėGabrielle Lynn McLemoreRyo IshiharaGaetano Antonio LanzaRyo KarakawaGaetano D. GargiuloRyo MatsuuraGaetano IerardoRyogo FuruhataGaetano IsolaRyoma MorigakiGaetano SantulliRyuichi OhtaGagliardi Anna RobertaRyuji HamamotoGaia BarisioneRyuta WatanabeGalileo EscobedoS. A. I. S. M. GhazaliGamal Mohamed El-SherbinyS. H. ShinGanapathi GanapathiS. RaymanGang ChenS. ShankarGang PengSaad SalemGaoussou TouréSaba KhaliqGarima DwivediSaba TabasumGarry KuanSabiha ParveenGary LoughranSabina Antonela AntoniuGary SteinmanSabina KrupaGaryfallia PeperaSabine BélardGaurav BaranwalSabine FriedrichGaurav GhagSabine Matou-NasriGaurav PandeySabyasachi DashGaurav V. SarodeSadaf JahanGeert MayerSadiya Bi ShaikhGelei XiaoSaeed AbdollahifardGenco GençdalSaeed M. BamatrafGennaro Mariano LenatoSaeed Ur RehmanGennaro MartucciSafa DamiatiGenwei ZhangSafwan OmranGeoff HackettSagar SohoniGeorg SprinzlSaid El ShamiehGeorge Alexandru MafteiSaid ShamiehGeorge BertsiasSaif KhanGeorge E. MacKinnonSaiful Effendi SyafruddinGeorge FotakopoulosSaiko SugiuraGeorge KontogeorgosSajad AhmadianGeorge L. HinesSakiko KanbaraGeorge PatrinosSakshi AroraGeorge Priya Doss CSakyi Samuel AsamoahGeorge SamanidisSally El‑ZahabyGeorge UmemotoSally I-Chun KuoGeorgiana NemetiSalvador García-DelpechGeorgios AndroutsopoulosSalvatore ChibbaroGeorgios BenetosSalvatore EvolaGeorgios D. MichailSalvatore FeoGeorgios E. PapadakisSalvatore Giovanni De SimoneGeorgios K. GlantzounisSalvatore Giuseppe CocuzzaGeorgios KatsarasSalvatore IaconoGeorgios KotzalidisSalvatore PetroneGeorgios LaliotisSalvatore ScognamiglioGeorgios PapathanakosSamantha PayneGeorgios S. MarkopoulosSamar TharwatGeorgios Sokratis ChatzopoulosSambit MishraGeorgios TagarakisSameer ChavanGeorgios VrakasSameera AbeyrathnaGerald FerrettiSamhati MondalGerald SchwerdtSaminder Singh KalraGerald TomkinSamira Samiee-ZafarghandyGerard Marshall RajSamrad MehrabiGerard PasternakSamrita DograGerardo CazzatoSamson G. KhachatryanGerardo FebresSamson N. DowlandGerardo Quintana-LópezSamuel E. SchrinerGereon SchälteSamuel X. ShiGermán CorredorSamuele VaccariGermán EbenspergerSan Ming WangGerman PerezSancar SerbestGernot FuggerSandeep SinghGernot KriegshauserSandeep ThannaGert NiebauerSandra BarbalhoGetúlio Pereira De OliveiraSandra Kalil BussadoriGholamreza HaqshenasSandra MastroiannoGhufran BabarSandra RodriguesGiacomo FarìSandrine LauranceGiacomo PapottoSang Hoon KimGiacomo Pietro VigezziSang Jin RheeGiada CrescioliSangamanatha Ankmnal VeerannaGiampaolo VettaSang-Ho KimGiampiero AvruscioSanja Popović-GrleGiampiero NeriSanjay GuptaGian Luca GraziSanjiv MahadevaGian Marco DumaSankaran DevimeenakshiGian Marco GhiggeriSanthosh Kumar PasupuletiGianfranco MitacchioneSantiago BallazGianluca L. ZingariniSantiago J. BallazGianluca TedaldiSara Al-GhadbanGianmaria SalvioSara C. ZapicoGianna WilkieSara MantiGianni OrofinoSara Moreno CamaraGiannis D. ParaskevopoulosSara NamvarGianpaolo PapaccioSara Salvador-MartínGideon CharachSara SarasuaGil SlutzkeySara SommarivaGilad YahalomSarah C. ShuckGilda SandriSarah Elizabeth TaylorGill A. Ten HoorSarah El-NakeepGino GrifoniSarah MartaindaleGioele CastelliSaral DesaiGiordana BettiniSaran LotfollahzadehGiorgia BussuSarath VijayakumarGiorgia ComaiSarhang HamaGiorgia TimonSarveen GajebasiaGiorgio BarberaSatheesh SengodanGiorgio BergaminiSathish ThirunavukkarasuGiorgio CalloviniSathishkumar V. E. Giou-Teng YiangSatilmis BilginGiovanna D’AndreaSatoru MunakataGiovanni CimminoSatoshi YamaguchiGiovanni CristalliSaud AlrawailiGiovanni DamianiSaul FrenkielGiovanni MeolaSaulius RočkaGiovanni MisseriSaulius RutkauskasGiovanni NanoSaurabh ChaturvediGiovanni PapaSaveg YadavGiovanni PerettoSaverio CosolaGiovanni RalliSaverio MuscoliGiovanni ScambiaSavio Domenico PandolfoGiovanni TarantinoSavvas LampridisGiovanni TrisolinoSayali MukherjeeGiovanni ZoccaliScott O’GradyGisela MezquidaSe Jin ParkGiselle Y. LópezSean Robinson SmithGiulia AbateSebastian FerriGiulia D’AurizioSebastian J. FlackeGiulia MidenaSebastian SchnaubeltGiuliana FavaraSebastian SzubertGiuliana MuzioSebastiano CiccoGiulio Francesco RomitiSebastien CelleGiulio VaraSebastien HascoëtGiuseppe BifulcoSeckin AkgulGiuseppe BroggiSeema MishraGiuseppe CaminitiSeema SikkaGiuseppe FenuSegun OlatinwoGiuseppe FerrandinoSegundo Nicolas SeclenGiuseppe GulloSeiichi YoshimotoGiuseppe LanzaSeiichiro MitaniGiuseppe MaccagnanoSeka LazareGiuseppe MagliuloSemih BaşçıGiuseppe MasciaSenija EminovićGiuseppe MercanteSenthil BhoopalanGiuseppe MurdacaSeogsong JeongGiuseppe NassoSeokchan EunGiuseppe SarpietroSeokhyun YoonGiuseppe SprianoSeong-Soo ChoiGiuseppe VaroneSepideh DarougarGiuseppina BarutelloSerge BrandGiuseppina CampisiSergey D. ArutyunovGiuseppina Maura FranceseSergey G. GavrilovGlenda Giorgia CaputoSergey KolomeichukGlenn LoboSerghei CovantevGloria BertoliSergi Fernandez-GonzalezGloria CosoliSergii BabichevGloria González-MedinaSergio De SalvatoreGniewko WięckiewiczSergio MalutaGodfrey M. RwegereraSergio SzachnowiczGoel ManviSeung Jin LeeGoh Choon FuSeung Uk LeeGokhan OzunerSeverin RodlerGonzalo Emiliano Aranda-AbreuSeydi YıkmışGonzalo R. Ríos-MuñozSeyed Ahmad Naseri-AlaviGopakumar GopalakrishnanSeyed Mostafa KiaGopal NambiSeyed Peyman ShariatpanahiGopal Nambi NambiShadi KatouGoran KrstacicShai FactorGoran SoderlundShailabh KumarGorka VuletićShalini DograGoto YoshikazuShamsah KazaniGowri ShankarShanlin KeGraciela Padilla-CastilloShannon KinneyGrady NelsonShantanu GuptaGrażyna BączekShaohua LuGrażyna JanikowskaShaojun LiuGregor ProsenShaoqing YuGregory E. SimonShao-Wen HungGregory M. HolmesSharad ChandraGrzegorz GrześkSharad PurohitGrzegorz JakubiakSharnil PandyaGrzegorz SzczȩsnySharon D. De MoraisGrzegorz ZielińskiShashank JariwalaGrzegorz ŻurekShatavisha DasguptaGuadalupe Gonzalez-OchoaSheba GoklanyGuadalupe Molina TorresShehwaz AnwarGuadalupe Rojas-SanchezSheikh AhmadGuang Yih SheuSheila VeroneseGudrun KoehlShelly R. McFarlaneGuennadi SaikoSheng-Dean LuoGuenther KahlertSheng-Der HsuGuido CaluoriSheng-Long DingGuido Di DonatoSheng-Mou HsiaoGuilaine BoursierShengshuai ShanGuilherme Cordenonsi Da FonsecaSherif Ashraf FahmyGuillem Pintos-MorellSherry MansourGuillermo Alberto KellerSherry S. CollawnGuillermo PlazaSherry X. YangGuillermo Til-PerezShi LiangGülbahar UstaoğluShibo YuGulzhanat AimagambetovaShigeki MatsubaraGunn AmmitzbollShihai LiGuobin XiaShih-Hsin KanGuoming ZhangShih-Ku LinGuoru ZhaoShih-Min HsiaGurdeep SinghShihyu ChangGurinder KumarShijie HeGurpreet Kaur AroraShilpi JainGuruprasad MahadevaiahShin Jun ParkGustavo Alencastro Veiga CruzeiroShin MatsuokaGustavo ArocaShinichi OhtaGwenllian TawyShinji HadanoH. Lester KirchnerShintaro FujiharaHaibo WangShintaro SengokuHaibo YangShirin SaberianpourHaidong LiShirley HillHailan FengShivanand PudakalakattiHaishu LinShiyu TangHaitham A. ElmarakebyShoba NarayanHaitham AbdelhakimShomona Gracia JacobHaitham JahramiShooka MohammadiHaiwen LiShravan GiradaHaixia JinShreesh SammiHak ChangShu HisataHalina WhiteShuangzheng JiaHalis SüleymanShu-Chun LinHaluk DamgaciogluShuhei YoshidaHamayak S. SisakianShu-Hui WenHamed JelodarShu-I. WuHamid MoghaddasiShuji OzakiHamidullah KhanShuji TachibanakiHammad A. GanatraShujiro HayashiHamoon ZohdiShu-Jui KuoHamza El HadiShulin ZhangHan Naung TunShun YamamotoHanae SaitoShun-Qing LiangHang SuShunsuke ShibaoHanish JainShuohong WangHan-Mou TsaiShuyang YangHanna MannellShuyu WangHans ChristiansenShwetabh SinhaHans G. DrexlerSiân E. HarrisonHans H. StassenSibusiso AlvenHans-Jonas MeyerSiddharth PahwaHaobijam BasantaSidharth MehanHaolun ShiSik NamgoongHaoran WeiSilvana BalzanHari Krishna NamballaSilvia De Miguel-BilbaoHarikumar RajaguruSilvia LakatošováHarold SniederSilvia LiuHaroon IqbalSilvia MascoloHarpreet KaurSílvia PinhãoHarshani WijerathneSilvia Reverté-VillarroyaHarshit ShahSílvia Rocha-RodriguesHasan MujtabaSilvio MaringhiniHasan ÖzdoganSilviu AlbuHasanain Faisal GhaziSimon Jasinski-BergnerHaseeb ChaudharySimon LyttonHashem AlsaabSimona Catalina StefanHassan Mohammad NaifSimona CimaHassan RanjbarSimona PetrucciHatem El-ShantiSimona SoveriniHatice Merve BayramSimona ZaamiHatim BoughanemSimone BattagliaHauke BuschSimone Cristina Soares BrandãoHauke ThomsenSimone Di PlinioHeather Leduc-PessahSimone GalloHéctor BurgosSimone GrassiHeiko EnderlingSimone La PadulaHeinz SchmidbergerSimone MorselliHelen AlmondSimone PernaHelena EnocssonSimone SerafiniHelena Fernández LópezSimran MaggoHelena Giannakaki-ZimmermannSina TaefehshokrHelena JoséSiqi ChenHelena MartynowiczSiranjeevi NagarajHelge Immo LehmannSitanshu S. SinghHelmut MairSivakumar NuvvulaHemang B. PanchalSiwei ZhangHemant Raj MutnejaSiyuan SuHemantkumar ChavanSkaidra ValiukevičienėHeming YaoSlobodan ObradovicHendrik BerthSmriti PandaHenriette HeinrichSnita SinukumarHenrik C. BäckerSofya P. KulikovaHenrik KöhlerSohini ChakrabortyHenrique PereiraSoledad García-Gómez-HerasHenry T. PengSolomon AdamsHenryk KasprzakSomchai AmornyotinHerbert Ryan MariniSonali NashineHermann KreyenbergSongkwan SilaruksHermizi HapidinSonia D’ArrigoHeying DuanSonia Di TellaHidehiro OkuSonja BidmonHidekazu HiranoSonu M. BhaskarHideo WadaSoo-Hyun SungHideyuki KawauchiSophia M. SchmitzHimangshu SonowalSoraya Paz MontelongoHimanshu JoshiSøren MøllerHind AlsharhanSorin BaracHipolito NzwaloSorin MihaliHiren K MewadaSotiria D. VrouvaHiroharu KataokaSotirios ApostolakisHiroki ToyodaSouichiro UshioHirokuni TakanoSounak SahuHiromitsu NegoroSousan ValizadehHironori IshiguchiSousana PapadopoulouHiroshi KurokiSouvick RoyHiroshi NoguchiSowmiya A. MoorthieHiroshi YokomichiŠpela ŠalamonHiroyoshi TakeuchiSreekanth Kumar MallineniHiroyuki TsuchiyaSridevi ChallaHiroyuki YashuiSrinivas NellimarlaHisham FansaStamatia TheodoridouHitoshi KuroseStanescu Ana Maria AlexandraHo Seok SeoStanisław HorakHo TsoiStanisław MlekoHolly L. PeayStanisław WołowiecHong In YoonStanley XuHong Wang FungStasja DraismaHonghe LiuStavroula BarbounakiHongli Sam GohStefan GeorgievHong-Tao LiStefan GroßHongwu JingStefan HeinrichHon-Yi ShiStefan HertlingHooman Soleymani MajdStefan J. LangHoon ParkStefan JovčićHorațiu Alexandru ColosiStefan Kurath-KollerHoria HaragusStefan NaydenovHoria PăunescuStefan Paul-AndreiHormos Salimi DafsariStefania BrozzettiHortensia Moreno-MacíasStefania PrincipeHossein EsmaeilzadehStefano CanosaHossein TaghizadehStefano CiardulloHoward LiStefano DastoliHrvoje JakovacStefano Di GirolamoHsiang-Yu LinStefano EramoHsien-Wei TingStefano Francesco CrinoHsin-An ChangStefano GhirardelliHsin-Chieh YehStefano Maria PriolaHsin-Yuan ChenStefano NobileHsueh-Ju LuStefano PalermiHsu-Heng YenStefano SaranHuapeng FengStefano SkurzakHubert Szymon GabryśSteffen GrautoffHugo Hyung Bok YooStephan AllgeierHugo MartinianoStephan AltmayerHuifen ZhouStephanie FinzelHuiyi CaoStephanie T. YerkovichHülya KarataşStephen InnsHungwon TchahStephen M. ModellHung-Yu HuangStephen P. BadhamHuriviades Calderón-GómezStergios BoussiosHusamettin VatansevSteve EllisHüseyin CanSteve GameiroHyeonjong LeeSteve Simpson-YapHyewon ChungSteven GiannottaHye-yon ChoStig SommeHyungon LeeStuart J. NethertonIan DeSouzaStuart McCluskeyIan G. DuncanStuart Paul AdamsIan M. ThornellSubhransu S. SahooI-Chien WuSubrata DebIdo FeferkornSucharat LimsitthichaikoonIdris LongSuchitra JoshiIeva StundienėSudarshan DayanidhiIgnacio Diez-VegaSudhakar JinkaIgnacio Navarro CuéllarSudhakar TummalaIgnacio TartaraSudhir Putty ReddyIgnatius Ryan AdriawanSudipta PanjaIgnazio CondelloSu-Fang LinIgor Age KosSujita Kumar KarIgor I. KireevSujith PereiraIgor P. KaidashevSumeet NayakIgor PetrusicSumesh ParatI-Hsi KaoSun Tae Ahnİkbal Çivi İnanlıSuneel KumarIkchan JeonSunetra SaseIkpe Justice AkpanSung Huang Laurent TsaiIkram SghaierSunga KongIlais Moreno VelásquezSunghoon ShinIlaria BertocchiSung-Soo KimIlaria GiambuzziSunil KarhadkarIlaria SimonelliSunil KumarIlaria Tocco TussardiSunny Chi Lik AuIldikó HorváthSunyoung ParkIlhami YukselSupat ChupraditImam AmmarullahSurajit BasakIman MomkenSurendra RajpurohitImmacolata PietraforteSurya KantImran Ali KhanSusan ColaceImran SiddiquiSusana B. BravoImtiaz AlìSusanne Fuchs-WinkelmannIna RissanenSusanne HousmansIndika Neluwa-LiyanageSushama SivakumarIndra J. DasSushil DubeyIndra RamasamySushil Kumar ShakyawarInés Aguinaga-OntosoSushil MishraInes KožuhSushruth EdlaInes PankonienSusmita BarmanInga Griskova-BulanovaSuzan CinarIngrid Fricke-GalindoSuzanne LewisIngrid HolmSven MånssonIoan ȘimonSvetlana A. LitvinovaIoan TileaSvetlana A. SmirnikhinaIoana Cristina DahaSvetlana EdenIoana MozosSvetlana V. GuryanovaIoana RotarSvetoslav G. NikolovIoana Roxana BordeaSvetoslav TodorovIoannis E. KougioumtzisSvjetlana MohrmannIoannis PapadiochosSwanand KoliIoannis TsamesidisSwati GargIoannis VentoulisSwati JainIonel Alexandru ChecheriţăSweety SamalIonuț Isaia JeicanSyed Ahmad Chan BukhariIonut LuchianSyed F. AhsanIppolito NotarnicolaSyed Furqan QadriIrena JekovaSyed Husain Mustafa RizviIrena TabainSyed NabilIrene Carrillo MurciaSyed Naqui KazimIrene SchiavettiSyed Shujaat Ali ZaidiIrfan MohamadSyed Thouheed AhmedIride Francesca CeresaSylvain RoyIrina GradinaruSyrine HizemIrina Iuliana CostacheSzymon SiecińskiIrina MateiSzymon SkoczyńskiIris LesserSzymon SzemikIrshad Ahmed HajamTadashi SofueIrwin K. WeissTadeja KuretIryna RusanovaTaha Koray ŞahınIsabel Cristina CéspedesTahmineh MokhtariIsabel Forner- CorderoTaiwo AremuIsabella SalzerTakafumi HosokawaIsis Gil-MiravetTakafumi KatoIsmail SyedTakafumi YanagisawaIsmet HortuTakahiro NemotoIsmini E. PapageorgiouTakahiro YamasakiIsrael Casado-HernándezTakanori ItoIsrael Pérez-TorresTakanori OhnishiIssam KoleilatTakashi IshinoIstván Adorján SzabóTakashi KameiItzhak OrionTakashi MatsushimaIvan A. LopezTakayasu MishimaIvan BanovacTakayuki MaruyamaIvan KopitovićTakayuki YoshidaIvan MalčićTakeji SaitohIvan Shterev DonevTakeji Takamura-EnyaIvana BabovicTakemi OtsukiIvana KholováTakenori HarunaIvana MilovanovićTakuma InagawaIvana ŠimićTal GazittIvana ŠpakováTal LevinsonIvana SutejTalida Georgiana CutIwona RzeszutekTamal SadhukhanIwona WertelTamara GulicIzabela ZakrockaTamara Nikolic TurnicIzhar Hyder QaziTamara SkoricJacek GagalaTamás BaranyaiJacek SzymańskiTamás BerkiJacob FreedmanTamás F. MolnárJacobus H. De WaardTamer SakrJacopo CiaffiTamer Y. SororJacopo FalcoTania ColasantiJacopo Francesco ImbertiTanja GonskaJacopo LisoniTanja KönenJacopo SabbatinelliTanya HeynsJacques-Emmanuel Galimard GalimardTao TongJad Adrian WashifTaobo HuJae Hoo LeeTara PerrotJae Yon WonTara RobertsJae-Ho LeeTara SudhadeviJae-Hyun KwonTarik Sqalli HoussainiJaejun LeeTariq MalikJafar JafariTaro OkayamaJagdeep KaurTaru SinghJagdeep PodichettyTassiane Cristina MoraisJagdeep RahulTatiana A. SemenenkoJagdish JoshiTatiana BerezinaJahanban-Esfahlan RanaTatiana S. KalininaJahanpour AlipourTatsuji MizukamiJaime Bustos-MartínezTatsuo ShimosawaJaime DelgadoTatsushi MutohJaime EscallonTatsuya FujikawaJaime Reyes-HernándezTatsuya YamazakiJakob DörlerTatyana BaltinaJakub Dalibor RybkaTauseef JamalJakub RuszowskiTayebeh KazemiJames BentleyTe-Chun ShenJames C. LeeTeddy Weifa YangJames LeighTedi BegajJames M. ChurchTeerasak DamrongrungruangJames PowersTe-Huei YehJames SolomonTeja Muralidhar KalidindiJames W. SonneTemidayo S. OmolaoyeJames WalterTeodora TelecanJan ChrastinaTeresa Ann GrenawaltJan Maciej ZauchaTeresa GrzelakJan MalikTeresa Mira GruberJan MuzikTeresa PerraJan PithaTereza DoušováJan StępniakTereza GabelićJan TrnaTero A. H. JarvinenJana KimijanovaTerry LichtorJana Lízrová PreiningerováTeru KamogashiraJana NemcováTeruya KomatsuJane K. NguyenTeruyo KidaJaneen R. JordanTessel RigterJanet KuhnkeTessho MaruyamaJanet WilliamsTetsuya KokabuJanez ZibertTetsuya OgawaJanne P. ThirstrupThach N. NguyenJannik PrasuhnThamir M. Al-KhlaiwiJaroslaw KwiecienThammanard CharernboonJarosław WaloryThanigaivelan KanagasabaiJasleen BirdiThaqif El KhassawnaJasmine LuzumThasinas DissayabutraJason A. SokolThayne KowalskiJauhar IqbalTheddeus Octavianus Hari PrasetyonoJavad TavakoliTheis Skovsgaard ItenovJaveed ShaikThemistoklis ExarchosJavier Carbajo-LozoyaTheocharis KoufakisJavier Menéndez-MenéndezTheodora WingertJavier Rodríguez VillanuevaTheodore W. LaetschJavier. CampaniniTheodoros AslanidisJawahar KalraTheodoros EleftheriadisJayanthi ParthasarathyThéophraste HenryJayapal Reddy MallareddyThijs Van Der ZijdenJean AmiralThomas J. DinzeoJean-Baptiste RiccoThomas KlingenhebenJean-Luc WautierThomas KrauseJeanMarie DelalandeThomas LiehrJean-Paul SoucyThomas MawhinneyJeewan ThapaThomas MohrJeff VictoroffThomas P. Van Der MeerJeffrey BotkinThomas T. H. WanJeffrey Che-Hung TsaiThomas TerrenceJeffrey D. DellaVolpeThomas ThomsenJeffrey Hugh GambleThury AttilaJeffrey LaurenceTiago Damas MartinsJeffrey Pradeep RajTiago DiasJelena Korac PrlicTiago MestreJelena KosticTianhua NiuJelica Grujić-MilanovićTihomir Georgiev-HristovJelle OverboschTihomir MustakovJennifer ChippsTijana KovačevićJennifer GuimbellotTim CrulJenny AtorfTim HulsenJens HöppnerTim ReynoldsJens KerlTim WalkerJeong-Yeol ParkTimothy E SweeneyJerónimo González-BernalTimothy HasenoehrlJerzy BiałeckiTimothy J. MeekerJerzy NarlochTin ČohadžićJesse C. KrakauerTina Tičinović KurirJessica Aijia LiuTina ŽagarJessica L. ChristensonTing LeiJessica RademacherTingwei ChangJesús Adrián LópezTinhinane FaliJesús García-CanoTivadar BaraJesus M. Martin-CamposTobias KielJi Wook JeongTobias RomeykeJiabao XuTodd RichardsJia-Ching ShiehTolga OlmezJiahui ChenTolga ZamanJiajia SongTomas SimurdaJiake XuTomáš StrašákJia-Lin FanTomasz FrączykJialin MengTomasz FuchsJianan ZhaoTomasz GabryśJiancheng QiTomasz HirnleJianfei XieTomasz KluzJianfeng LiTomasz P. KonopkaJiangang ChenTomasz RechcińskiJiangbin WuTomasz RusakJianghua ChenTomasz TokarekJianglei DiTomasz UrbanekJiangong CuiTomislav TostiJianzhen XuTommaso CaiJiaqiang HuangTommaso FabrizioJiaqiang ZhangTomohiro UdagawaJiawei XiangTorbjörn LedinJiayi PeiTorsten K. MalmJiayue QinTorsten KrayaJie GuTorsten MundtJie HuTorsten SteinbrunnJie LeeToshiki KaiharaJie YangTraian DumitraşcuJien Jiun ChenTri Indah WinarniJih-Yang KoTristan LaneJijia WangTristan WagnerJill GoldmanTse-Shao ChangJimei WangTsung-Li LinJin WangTsu-Te YehJin ZhangTsutomu NakashimaJina OhTsutomu YasukawaJing HanTsvetelina BatsalovaJing ZhouTudor DruganJingjing TangTudor Sorin PopJingjing WuTudoran CristinaJinlei ShenTugba Bagci-OnderJinsheng LuTuo MengJinwu WangTurcu-Stiolica AdinaJitendra KanshanaTyler J. TitcombJitendtra Kumar Singh PariharTze-Chien ChenJitse P. Van DijkUehara TomokoJiyoun SongUenis TannuriJo Anne StrattonUgo CarraroJoana MesquitaUgur CavlakJoanna Hermanowicz-SalamonUlf C. SchneiderJoanna KapustaUlf SchminkeJoanna KosackaUlisses Moreno-CelisJoanna LandmesserUmakant SahuJoanna M. ZakrzewskaUmar ShafiqueJoão Gaspar-MarquesUmberto AnceschiJoão M. SantosUmer SaleemJoão Pinto-De-SousaUri KaplanJoão Sequeira AlvesUrko M. MarigortaJoaquim CaladoUrna KansakarJoaquim Murray Bustorff-SilvaUtkarsh KohliJoaquim Soares Do BritoUttam SatyalJody A. RuleVahideh RabaniJoel W. HughesVaideesh ParasaramJohad F. KhouryValentin M. VetterJohanan Christian PrasanaValentin VarlasJohann SellnerValentina CalòJohanna KirchbergValentina FavalliJohanna RommensValentina ManzoJohannes WachValentina OpancinaJohari Yap AbdullahValentina SancisiJohn A. Albert CyrValentina ValleJohn A. DeliaValeria AlbanoJohn GriniatsosValeria CoviltirJohn HancockValeria RaiaJohn O. OnukwuforValeria Soto-MendozaJohn P. VavalleValeria ViscoJohn RoshanValērija GromaJohnny PaduloValerio CaputoJo-Hsuan WuValery V. LikhvantsevJolanta Nawrocka-RutkowskaValeska HofmannJolanta RzymowskaValliappan RamanJonas Michel WolfVan Tran ThuanJonathan ClarkVanesa BlázquezJong Cheol ShinVanesa BochkezanianJonghwa JangVanessa MoreiraJonnathan Guadalupe Santillán-BenítezVanja KaliternaJoo-hun ParkVanja Martinovic JovicevicJoon Hyuk ChoiVarun GuptaJordan HalseyVasile PotopJorddy Neves CruzVasile-Bogdan HalatiuJörg MarotzVasileiou PanagiotisJorge Gonzalez-GutierrezVasilije StambolijaJorge H. LeitãoVasilios PergialiotisJorge MartinsVasudev BallalJorge N. R. MartinsVedat EljeziJorge RoaVeenesh SelvaratnamJørn Bolstad ChristensenVeerupaxagouda PatilJose A. Lopez-EscamezVera GuttenthalerJosé A. RizzoVera LarinaJosé AlfieVera Sa-IngJosé Ángel ArmengolVeronica Alexandra AntipovaJosé Antonio Garrote-AdradosVeronica Elena TrombitasJose Antonio González CorreaVeronica Perez-De La CruzJosé Antonio Morales-GonzálezVeronika HuntosovaJosé Antonio PintoVesna Vranić MandušićJosé Augusto SimõesViacheslav VoroninJosé Bruno Nunes SilvaVibhu Krishnan ViswanathanJosé Daniel SubielaVibor MilunovićJose Fernández-SáezVicenç LeónJosé Francisco Muñóz-ValleVicente Bertomeu-GonzálezJosé Francisco Rodríguez-VázquezVicente Jose Diago-AlmelaJosé Hernández-TorrucoVictor B. LoschenovJose IglesiasVictor Ivanovich SeledtsovJose L. Garcia-SerranoVictor MarinhoJosé López CastroVictor Vlad CostanJosé M. FradeVictoria GaribJosé Maria LassoVictoria LópezJosé Miguel Rivera-CaravacaVictoria M. PrattJose PalaciosVictoria WorkmanJosé Pedro MartinhoVida Gavrić LovrecJosé-Ángel Hernández-RivasVidya RattanJose-Angel Pérez RiveraVijay AvinJosef RuzekVijay GanjiJosef SmolleVijay IndukuriJosefa Gonzalez-SantosVijayalakshmi PoreddiJosefina CernadasVijayaprakash SuppiahJosé-Juan Pereyra-RodriguezVikrant RaiJoseph AttiasViktor ČernýJoseph J. DisaViktor ChrobokJoseph KauerViktor DomisloviĆJoseph M. RosenViktória FisiJoseph M. RutkowskiViktoria RajtukovaJoseph MalinziVilas NasareJoseph NassifVilborg PalsdottirJoseph NdikaVinay PitchikaJoseph Olusesan FadareVinayak M. JoshiJoseph P. DewulfVincent H. J. Van Der VeldenJoseph RaffettoVincenza LaforgiaJoseph RinehartVincenzo CalabreseJoshua ChewVincenzo CastiglioneJosip A. BorovacVincenzo FiorentinoJosip MadunićVincenzo GrassiaJosipa KernVincenzo NuzziJoško ŠodaVincenzo SavarinoJosue MartosVincenzo ToscoJovana Joksimovic JovicVioletta Opoka-WiniarskaJoydeep ChakrabortyViorel JingaJrhau LungViorel RaducanJuan AyalaViorela Mihaela CiorteaJuan Carlos López-GarcíaVirgilijus UlozaJuan Francisco Fritche-SalazarVirginia LiberiniJuan Garduño-EspinosaVirginie Monnet-CortiJuan Gustavo Vázquez-RodríguezVishakha Anand PawarJuan José LarrañagaVishnu Muthuraj KumarasamyJuan Luis Rodríguez HermosaVishnu Priya MuraliJuan Luis Sánchez GonzálezVishnu TripathiJuan M. Fernández-CostaVishnuprabu Durairaj PandianJuan Pedro Martínez RamónVitalii LunovJuan Vega EscañoVito Andrea CapozziJudith C. KreutzmannVittoria Di MauroJudith Catella-ChatronVittorio AprileJuergen ReichardtVittorio CherchiJuhani MaattaVlad GorduzaJuhra ChristianVlad PadureanuJulia LorenzoVlad PorumbJulian S. RechbergerVladimir A. ShvartzJuliana De Oliveira CostaVladimir M. ShipulinJuliana SchwaabVladimir PisarevJulie LapointeVladimir TrkuljaJulie SwartzendruberVladimir VladimirovJulieanne P. SeesVolkan ÜlkerJulien AncelVolker EulenburgJulien CabatonVrushank BhattJulien WilsVrutant ShahJuliet Haarbauer-KrupaVui King Vincent-ChongJulijus BogomolovasWael SaadeJulio DuarteWaleed K. AbdulsahibJulio Enrique Castañeda-DelgadoWalter ColangeliJulio GarciaWalter J. FlapperJumpei SaitoWalter L. StrohmaierJun HarumaWalter R. SchummJun Hui JeongWan Aliaa Wan SulaimanJun LuWan Nordiana RahmanJun Neng RoanWang XiChiJun YanWang Zheng-XinJun Yi SimWanguo LiuJunaid AhmadWankun XieJunaidi KhotibWarawut ChaiwongJunfei ZhaoWassan NoriJung SunwooWathik AlsalimJung-Joon ChaWayne Owen MilesJungtae LeeWei FengJunhao ZhuWei ShaoJunichi SaitoWei SunJunichi ShimizuWei Wen LimJun-Il YooWei-Chieh HuangJunrui ChengWei-Chieh LeeJure UrbancicWeichih ChenJusaku MinariWei-Chun LinJu-Seop KangWeihong ChenJustas ZilinskasWeijian BeiJuste KaciulyteWeiren LuoJustin AronoffWei-Ti ChenJustin M. RichnerWeiwei CuiJustyna Chojdak-ŁukasiewiczWeiyu LiJustyna Cwajda-BiałasikWeiyun ShiJustyna Jolanta MiszkiewiczWen LinJustyna Opydo-SzymaczekWenchen JiJustyna SikoraWenchen WuJu-Yi ChenWen-Chung LiuK. N. ChethanWenfei SunK. S. AnandhWenguang YangKaare GautvikWenjun KouKa-Chun WongWen-Tyng LiKai CaiWenwen TangKai Cheng HsuWenzheng JiangKai JinWerner SchrothKai LiWibke Müller-SeubertKai ShuWieland ElgerKaikai XuWilliam David DotsonKai-Wen HsuWilliam Y. RaynorKaiye GaoWilly HauzerKalliopi PlatoniWładysław LasońKamalesh ChakravartyWojciech EliaszKamil BarańskiWojciech FroelichKamil NelkeWojciech GawęckiKamonwon IenghongWojciech JurczakKamran ShaukatWojciech SkrobotKannan SridharanWojciech SzlasaKara GavinWolfgang A. WetschKaram M. ObeidWolfgang RottbauerKaren L. SternWolfgang SenkerKaren SuetterlinWolfgang ThaissKaren Villar ZarraWoo-Baek ChungKarim FifelWoonhyeok JeongKarin PfisterWoori KimKarl-Arne JohannessenWullianallur RaghupathiKarl-Heinz WidmerXando Díaz-VillamarínKarmen StankovXavier Bofill-de RosKarol WiśniewskiXavier MoratóKarolina ChilickaXi LiuKarolina KossakowskaXiang WangKarolina OsowieckaXiangrong ZhengKarthik RamamurthyXiaodong ZhouKartik DhadukXiaofei ChenKartika Afrida FauziaXiaofei JiKassiani TheodorakiXiaohu HuangKatarina ItrićXiaojie SunKatarina SwahnbergXiaoju HuKatarina VukojevicXiaoming ZhengKatarzyna Beata CywkaXifan MeiKatarzyna KaczyńskaXimena Alejandra León-RíosKatarzyna KorybalskaXin LiKatarzyna Plagens-RotmanXin SunKatarzyna RygielXin WangKatarzyna ZaorskaXin WeiKate HsuXin ZhangKaterina KaraivazoglouXinan WangKaterina KatsarakiXinghua WangKatharine HardingXinlei LiKatherine A. B. KellettXinli LiKatherine AlpaughXinlu DingKatherine J. E. SattlerXuan LiKathryn AlsopXuanang XuKatianne Howard SharpXucheng HouKatja S. C. GollischXudong ZhangKatrien VermeireXue-Bo JinKatrin Schaper-GerhardtXueying WangKatrin ScheinemannY. O. DavydovaKatrina E. FurthYacine OuahchiKatrina O’HalloranYafei WangKatsuya KitamuraYahya SohrabiKatsuyuki TanabeYan Chai HumKavitha S. RaoYan GengKay Choong SeeYanfang JiangKazuaki ImaiYang XiaoKazuhide Shaun OkudaYang YangKazuhiro P. IzawaYang ZhangKazuki HiraoYangpeiwei HuangKazunori NagasakaYannick Fabian DiehmKazunori NozakiYanning ZuoKee Huong LaiYanwen WangKeigi FujiwaraYanzhe XuKeigo IkedaYao TongKeittisak SuwanYaolung ChangKeivan ShariatmadarYaping WangKellie ArcherYasmina Abd-ElhakimKelsey CookYasuhiro TakahashiKenichi SudaYasuko FujitaKenji HayashidaYasunari MatsuzakaKenji SatouYasushi NishiiKenneth A. AndreoniYasushi TsujiKenneth C. BilchickYasushige ShinguKenneth HandelmanYasuyuki TokinagaKenta MurotaniYawei LiKenza E. BenzeroualYazeed BarghouthyKeti VitanovaYegor TryliskyyKeun-Yeong JeongYen Y. TanKevin FonsecaYen-Lin LiuKevin M. SullivanYen-Wen ShenKevin StaatsYeon-Ha KIMKevin T. BainYeunjoo E. SongKevin Y. YangYi Hui WuKeyla Vargas-RománYi LuanKhaled EmaraYiannis VasilopoulosKhaled Q. Al HamadYi-Chiung HsuKhalid M. AlmasYifei XueKhalid Walid BibiYifeng QiKhalil KarimiYi-Hsuan HsiaoKhawla S. Al-KurayaYi-Hsuan WeiKhayriyyah Mohd HanafiahYıldıray YalmanKhristine Joy C. AntiguaYiming TangKhushboo MunirYin LiKian KaniYin Ping WongKibum KimYinan JiangKiichi TagoYing LiuKi-Kwang OhYing-Chun ChienKilian RücklYinglun ZhanKimberly H. WoodYirong ChenKimberly S. ToppYi-Shin HuangKimon RungeYoav KohnKin Yin CheungYoga YuniadiKinga GawelYogish Subraya KamathKinga TurzoYoji KishiKiran SandhuYokrat TonKiran UpadhyayYong An ChungKishore BharathyYong WangKishu RanjanYong X. GanKitanaka JunichiYonggang ZhangKlára DaďováYong-Hak KimKlaudia DopytalskaYonghe MaKlaus H. HoffmannYongquan ZhouKnut TaxbroYool LeeKoichi TairaYoon Soo ChangKomsun SuwannarurkYoshiaki ZaizenKonosuke NakajiYoshihiro FukumotoKonstantin BergmeisterYoshimitsu GotohKonstantinos A. GatzoulisYoshinori ShiraiKonstantinos G. KyriakoulisYoshiro ItoKonstantinos KypriotisYoshitaka ShimotaiKonstantinos N. AnyfantisYoshiyuki MishimaKorakot ApiratwarakulYoshiyuki NakamuraKornelia ZarębaYoshiyuki ShioyamaKota KodamaYoufeng ZhuKourosh SheibaniYoug Raj ThakerKoya OzawaYoun Joung ChoKranti BhavanaYounes JabraneKresimir DolicYoung KoKrishna Kishore UmapathiYoung Suk KwonKrishnaji R. KulkarniYoung-Joon JunKrista Delviks-FrankenberryYousif Al MashhadanyKristen GuynesYoussra Al-HilalyKristen J. NicholsonYuan HuKristen M. TecsonYuanyuan ShiKristina KuhbandnerYubin KangKristoffel R. DumonYue GuanKristoffer StrålinYuehui YuKrisztina KelemenYuejiao XianKrisztina TarYuexin XuKrystian WochnaYuhei MatsudaKrzysztof CzyżewskiYuichi AdachiKrzysztof LetachowiczYuichiro HatanoKrzysztof PałganYuichiro YoneokaKrzysztof SzyfterYu-Jer HsiaoKrzysztof ZielinskiYuji ShimizuKsenija Rener-SitarYuka KotozakiKuang-Tai KuoYuki TabataKuan-Hao TsuiYukihiro SaitoKuanJung ChenYuko HaradaKuldeep ShahYuliya I. RaginoKun LiYuliya SemenovaKun LiuYun PanKun QinYun WangKun WangYun ZhuKundlik GadhaveYun-Chen ChangKun-Eek KilYundi FengKun-Hua LeeYungi KimKuo-Ting SunYuning ChenKurt A. MelstromYuqin QiaoKurt ChristensenYurii ChepurnyiKurt SvärdsuddYuriy L. Vasil’evKushal GandhiYu-Sheng YangKwon Hui SeoYusof KamisahKyle BurghardtYusra Habib KhanKyo Chul KooYusuke EndoKyoung Sik ParkYusuke SekinoKyung Rae KoYuta TsubouchiKyungjin KimYutaka ImamuraKyung-su KimYutaka OwariL. Morrison ClaireYuting WangL. RuffiniYu-Wei LiuLachlan KentYves HélouryLaffranchi MattiaYves LaumonnierLai-San WongZack Y. ShanLara ReichertZaher BahouthLarisa AnghelZahra ShahsavariLarisa Prpić-MassariZainub JoomaLars NorgrenZala LuznikLaszlo Barna IantovicsZbârcea Cristina ElenaLászló Csaba MangelZelic KsenijaLászló MódisŽeljko AntićLászló NagyZeyu LuoLászló SzabóZhen WangLatha PalaniappanZhengwu PengLatifa Rbah-VidalZhenhong WangLaukhtina EkaterinaZhenjie LiuLaura C BerumenZhenqi JiangLaura CastellettiZhibin LvLaura CobusZhibin YinLaura ConejeroZhihao ChenLaura CortesiZhimin TaoLaura DownieZhipeng HuLaura LindsayZhongbao ZhouLaura María CompañZihao OuLaura MitreaZoltán HeroldLaura Mouriño-AlvarezZsolt KovacsLaura PezzoliZsuzsa Máthéné SzigetiLaura RăducuZsuzsanna KahánLaura Wallard



